# The uterine epithelial loss of Pten is inefficient to induce endometrial cancer with intact stromal Pten

**DOI:** 10.1371/journal.pgen.1007630

**Published:** 2018-08-24

**Authors:** Xiaohuan Liang, Takiko Daikoku, Jumpei Terakawa, Yuya Ogawa, Ayesha R. Joshi, Lora H. Ellenson, Xiaofei Sun, Sudhansu K. Dey

**Affiliations:** 1 Division of Reproductive Sciences, Perinatal Institute, Cincinnati Children’s Hospital Medical Center, Cincinnati, Ohio, United States of America; 2 College of Veterinary Medicine, South China Agricultural University, Guangzhou, China; 3 Institute for Experimental Animals, Kanazawa University Advanced Science Research Center, Kanazawa, Ishikawa, Japan; 4 Department of Pathology and Laboratory Medicine, New York Presbyterian Hospital-Weill Medical College of Cornell University, New York, New York, United States of America; Cleveland Clinic Genomic Medicine Institute, UNITED STATES

## Abstract

Mutation of the tumor suppressor *Pten* often leads to tumorigenesis in various organs including the uterus. We previously showed that *Pten* deletion in the mouse uterus using a *Pgr-Cre* driver (*Pten*^*f/f*^*Pgr*^*Cre/+*^) results in rapid development of endometrial carcinoma (EMC) with full penetration. We also reported that *Pten* deletion in the stroma and myometrium using *Amhr2-Cre* failed to initiate EMC. Since the *Pten*^*f/f*^*Pgr*^*Cre/+*^ uterine epithelium was primarily affected by tumorigenesis despite its loss in both the epithelium and stroma, we wanted to know if *Pten* deletion in epithelia alone will induce tumorigenesis. We found that mice with uterine epithelial loss of *Pten* under a *Ltf-iCre* driver (*Pten*^*f/f*^*/Ltf*^*Cre/+*^) develop uterine complex atypical hyperplasia (CAH), but rarely EMC even at 6 months of age. We observed that *Pten*^*f/f*^*Pgr*^*Cre/+*^ uteri exhibit a unique population of cytokeratin 5 (CK5) and transformation related protein 63 (p63)-positive epithelial cells; these cells mark stratified epithelia and squamous differentiation. In contrast, *Pten*^*f/f*^*Ltf*^*Cre/+*^ hyperplastic epithelia do not undergo stratification, but extensive epithelial cell apoptosis. This increased apoptosis is associated with elevation of TGFβ levels and activation of downstream effectors, SMAD2/3 in the uterine stroma. Our results suggest that stromal PTEN via TGFβ signaling restrains epithelial cell transformation from hyperplasia to carcinoma. In conclusion, this study, using tissue-specific deletion of *Pten*, highlights the epithelial-mesenchymal cross-talk in the genesis of endometrial carcinoma.

## Author summary

Endometrial cancer is highly prevalent gynecological cancer in the United States. *Pten* is the most commonly mutated gene in endometrial carcinoma originating in the epithelium. Previous studies focused on PTEN’s role in epithelial growth regulation. Here we show that in addition to *Pten* mutation in the epithelium, its mutation in the stromal compartment is critical for the initiation and progression of endometrial carcinoma. We present evidence that while loss of *Pten* function in both uterine epithelia and stroma results in rapid development of endometrial carcinoma, its loss in epithelial cells leads to endometrial hyperplasia, but not carcinoma. Our findings highlight the critical role of stromal PTEN in the transformation of hyperplasia to carcinoma and stromal TGFβ appears to play a role in preventing this transformation. This study reveals a previously unidentified role of PTEN in influencing the microenvironment in the uterus for the initiation and generation of endometrial carcinoma.

## Introduction

Endometrial carcinoma (EMC) is the most common cancer of the female reproductive organs in the United States. In 2017, about 60,000 new cases were diagnosed and about 11,000 deaths occurred related to EMC in the US [1, 2]. EMC has been categorized into two major types: type I endometrioid cancers are focused in the endometrial gland cells, and type II non-endometrioid cancers are often of serous morphology. Type I represents approximately 85% of EMCs in which *Pten* is commonly mutated. Other than endometrial cancer, *Pten* mutations are also evident in endometrial hyperplasia [3–5]; hyperplasia is a well-established precursor lesion of EMC [6]. The understanding of divergence between hyperplasia and cancer is of clinical significance. On one hand, faulty diagnosis of complex atypical hyperplasia (CAH) may lead to hysterectomy [7], a non-reversible procedure that negatively impacts women seeking to preserve fertility. Alternatively, diagnosis at early stage of hyperplasia may prevent progression to carcinoma. Although stromal invasion and histological changes are considered diagnostic standards of EMC [8], identification of the biomarkers for early stage carcinomas and the mechanism underlying cancer progression are greatly needed.

*Pten* homozygous null mice are embryonic lethal. Therefore, *Pten* heterozygous mice are widely used for cancer studies [6]. *Pten* heterozygous females show atypical endometrial hyperplasia phenotype, with 20% developing cancer. Using the *Cre-loxP* system and *Pgr-Cre* driver, we previously showed that *Pten*^*f/f*^*Pgr*^*Cre/+*^ mice with endometrial *Pten* deletion develop epithelial carcinoma as early as one month of age with *Pten* loss in major uterine cells [9]. To study roles of *Pten* in different uterine cell types, we created mice with *Pten* deletion specifically in the stroma and myometrium using *Amhr2-Cre* driver [10]. *Pten*^*f/f*^*/Amhr2*^*Cre/+*^ females showed no EMC; instead myometrial cells transformed into adipocytes [11]. Taken together, these findings suggest epithelial origin of this pathology. We thought that the epithelial origin of EMC could be tested if only epithelial-specific loss of *Pten* is induced in the uterus, disrupting the cross-talk between the stroma and epithelium to initiate EMC and its progression.

To address this issue, an efficient *Cre* mouse line is necessary to specifically delete epithelial genes. *Pten* was conditionally deleted in the epithelium using *Wnt7a-Cre*, but the mutant pups died around 10 days of age [11]. Conditional deletion of *Pten* using *Sprr2f-Cre* met with failure because of brain cancer and limited life span [11, 12]. To generate a mouse line with *Cre* activity specifically in the adult uterine epithelium, we generated a mouse line expressing codon-improved *Cre* (*iCre*) under a *Lactoferrin* (*Ltf*) promoter. By crossing with *LacZ* reporter mice, we showed that the *Ltf*-driven *iCre* expression exhibits robust Cre activity in uterine luminal and glandular epithelia beginning at puberty [13]. In contrast to *Cre* expression driven by promoters of *Pgr*, *Amhr2*, and *Wnt7a* that occur before or right after birth, Cre activity driven by *Ltf* promoter is activated with the beginning of estrous cycle [13].

In this study, using *Ltf*^*Cre/+*^ mice, we established the mouse model with uterine epithelial-specific *Pten* deletion by crossing with *Pten*^*f/f*^ mice. Surprisingly, *Pten*^*f/f*^*Ltf*^*Cre/+*^ females rarely develop EMC, but show epithelial CAH. We also found that *Pten*^*f/f*^*Ltf*^*Cre/+*^ females do not readily form stratified epithelial layers which are prevalent in *Pten*^*f/f*^*Pgr*^*Cre/+*^ uteri. *Pten*^*f/f*^*Pgr*^*Cre/+*^ epithelial layers show the presence of CK5, a stratified epithelial cell marker, and CK8 that is primarily expressed in simple epithelial cells. CK5 positive cells are located between CK8 positive epithelia and stroma, and the two populations of CK5 and CK8-positive cells are mutually exclusive in *Pten*^*f/f*^*Pgr*^*Cre/+*^ epithelia. p63 is critical for initiation of epithelial stratification [14], and has been identified as a prognostic marker in multiple cancers [15, 16]. Interestingly, p63 is expressed in CK5 positive cells in *Pten*^*f/f*^*Pgr*^*Cre/+*^ epithelia. We further identified TGFβ, which represses stratification of epithelium [17], is downregulated in the *Pten*^*f/f*^*Pgr*^*Cre/+*^ stroma as compared to that in *Pten*^*f/f*^*Ltf*^*Cre/+*^ mice. The differential phenotypes between *Pten*^*f/f*^*Pgr*^*Cre/+*^ and *Pten*^*f/f*^*Ltf*^*Cre/+*^ mice highlight the crucial role of stromal microenvironment and stromal-epithelial interactions in EMC progression.

## Results

### Conditional deletion of *Pten* in uterine epithelia leads to CAH without generation of EMC

**Fig 1:**
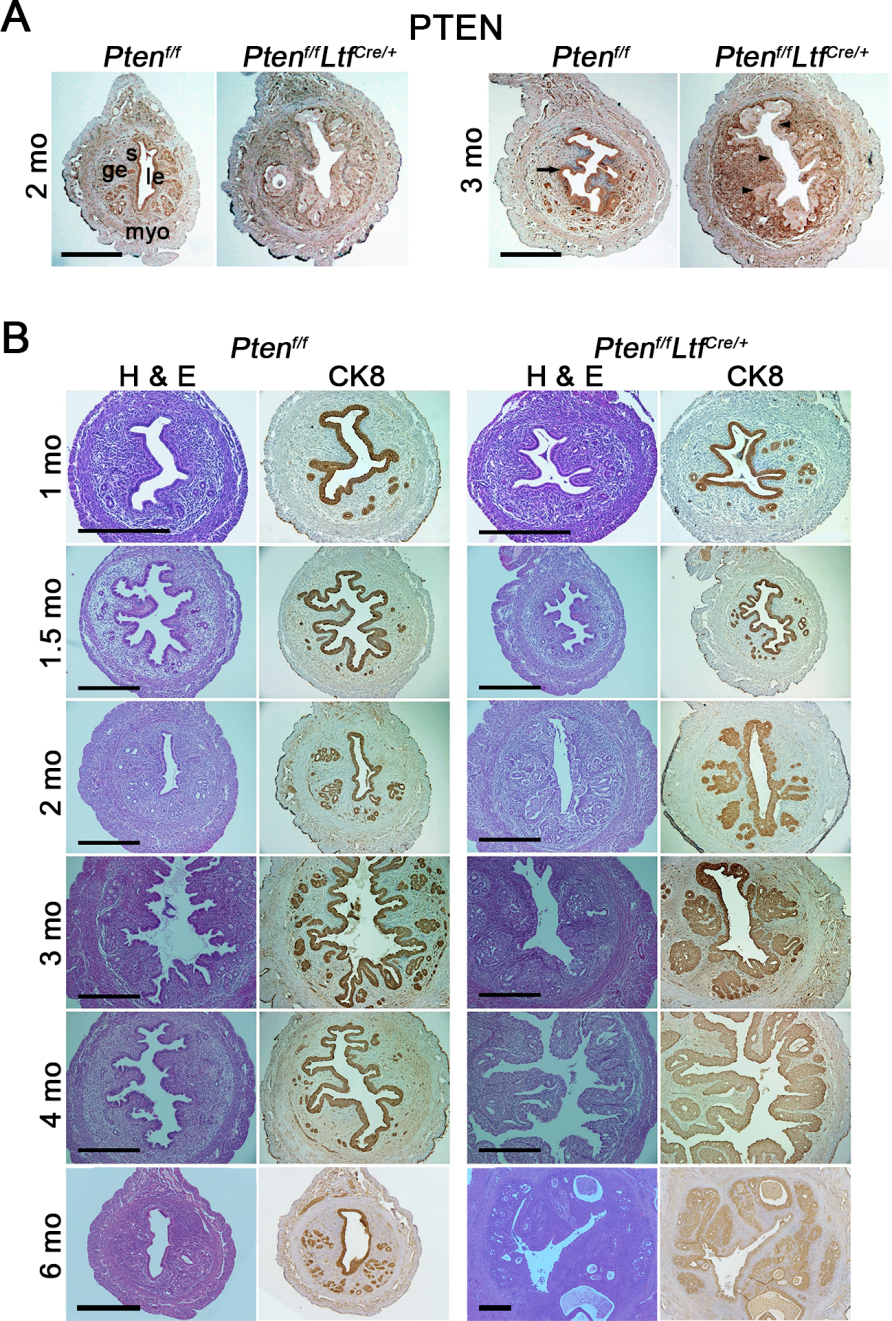
*Pten*^*f/f*^*Ltf*^*Cre/+*^ uteri show complex atypical hyperplasia (CAH). A, Immunohistochemistry of PTEN in 2 and 3-month-old *Pten*^*f/f*^ and *Pten*^*f/f*^*Ltf*^*Cre/+*^ uteri. *Arrow*, PTEN positive *Pten*^*f/f*^ epithelium. *Arrowheads*, *Pten*^*f/f*^*Ltf*^*Cre/+*^ epithelium with *Pten* deletion. B, CK8, Hematoxylin and eosin staining in *Pten*^*f/f*^ and *Pten*^*f/f*^*Ltf*^*Cre/+*^ uteri at different ages. Representative results from three individual mice are presented for each group. Scale bars, 400 μm. *le*, luminal epithelium; *ge*, glandular epithelium; *s*, stroma; *myo*, myometrium.

In *Pten*^*f/f*^*Ltf*^*Cre/+*^ mice, genomic deletion of *Pten* begins at 1 month of age (S1A Fig). By 2 months of age, rare PTEN positive signals, if any, are observed in the uterine epithelium (Fig 1A, S1B Fig). Notably, levels of PTEN expression in *Pten*^*f/f*^*Ltf*^*Cre/+*^ stroma are upregulated as compared to those in *Pten*^*f/f*^ mice at 3 months of age. We examined whether epithelial deletion of *Pten* produces EMC. Pathological analysis shows that *Pten*^*f/f*^*Ltf*^*Cre/+*^ uteri exhibit normal histology by 1.5 months old, but most of *Pten*^*f/f*^*Ltf*^*Cre/+*^ uteri start developing CAH from 2 months of age. By 4 months, only 2 of 8 (25%) mice show focal myometrial invasion (Table 1). *Pten*^*f/f*^*Ltf*^*Cre/+*^ mice at 6 and 12 months of age also show atypical glandular hyperplastic epithelia showing medium to large cysts with fluid retention. This glandular hyperplasia perhaps predisposed to carcinoma, but no epithelial invasion to the myometrium was evident (S2 Fig). Further analysis shows that PTEN expression patterns are comparable in *Pten*^*f/f*^*Ltf*^*Cre/+*^ mice with or without myometrial invasion (S3 Fig). Detailed pathological analyses at different ages is presented in **Table 1**. The results show that progression of EMC is dramatically retarded in *Pten*^*f/f*^*Ltf*^*Cre/+*^ mice as compared to that in *Pten*^*f/f*^*Pgr*^*Cre/+*^ uteri [9], suggesting that stromal *Pten* suppresses transformation of CAH to EMC.

**Table 1:** Pathological analysis of *Pten*^*f/f*^*Ltf*^*Cre/+*^ uteri.

Age (month)	Genotype	No. of mice examined	Uterine histology	No. of CAH (%)
**1**	*Pten*^*f/f*^	5	No pathology	0 (0%)
	*Pten*^*f/f*^*Ltf*^*Cre/+*^	5	No pathology	0 (0%)
**1.5**	*Pten*^*f/f*^	6	No pathology	0 (0%)
	*Pten*^*f/f*^*Ltf*^*Cre/+*^	5	No pathology	0 (0%)
**2**	*Pten*^*f/f*^	5	No pathology	0 (0%)
	*Pten*^*f/f*^*Ltf*^*Cre/+*^	8	CAH	6 (75%)
**3**	*Pten*^*f/f*^	6	No pathology	0 (0%)
	*Pten*^*f/f*^*Ltf*^*Cre/+*^	11	CAH	11 (100%)
**4**	*Pten*^*f/f*^	5	No pathology	0 (0%)
	*Pten*^*f/f*^*Ltf*^*Cre/+*^	8	CAH^a^	8 (100%)
**≥6**	*Pten*^*f/f*^	7	No pathology	0 (0%)
	*Pten*^*f/f*^*Ltf*^*Cre/+*^	7	CAH^b^	7 (100%)

^a^Two mice with CAH showed myometrium invasion.

^b^Five mice with glandular hyperplasia with predisposition to carcinoma.

### Conditional *Pten* deletion in the epithelium triggers AKT/mTORC1 signaling and COX-2 expression

PTEN, a phosphoinositide 3-phosphatase, metabolizes phosphatidylinositol 3,4,5-trisphosphate (PIP_3_) [18, 19], and suppresses AKT activation [20, 21]. As expected, AKT activation markedly increases in *Pten*^*f/f*^*Ltf*^*Cre/+*^ uterine epithelia at both 2 and 3 months of age (S4A Fig). Previously, we reported that mTORC1 is a downstream target of PTEN/AKT signaling in *Pten*^*f/f*^*Pgr*^*Cre/+*^ uteri [22]. In *Pten*^*f/f*^*Ltf*^*Cre/+*^ mice, mTORC1 activation is upregulated in epithelia at both 2 and 3 months of age, as evident from elevated levels of phosphorylated ribosomal protein S6 (pS6), a downstream effector of mTORC1 (S4B Fig). Heightened COX-2 expression and mTORC1 activity exacerbate EMC in *Pten*^*f/f*^*Pgr*^*Cre/+*^ uteri [22]. In *Pten*^*f/f*^*Ltf*^*Cre/+*^ uteri, COX-2 expression is induced in *Pten*^*f/f*^*Ltf*^*Cre/+*^ epithelia (S4C Fig). Western blotting results confirmed upregulated levels of p-AKT and pS6 in 2-month old *Pten*^*f/f*^*Ltf*^*Cre/+*^ uteri (S5 Fig).

### p-AKT, pS6, and COX-2 are differentially expressed between *Pten*^*f/f*^*Ltf*^*Cre/+*^ and *Pten*^*f/f*^*Pgr*^*Cre/+*^ uteri

**Fig 2:**
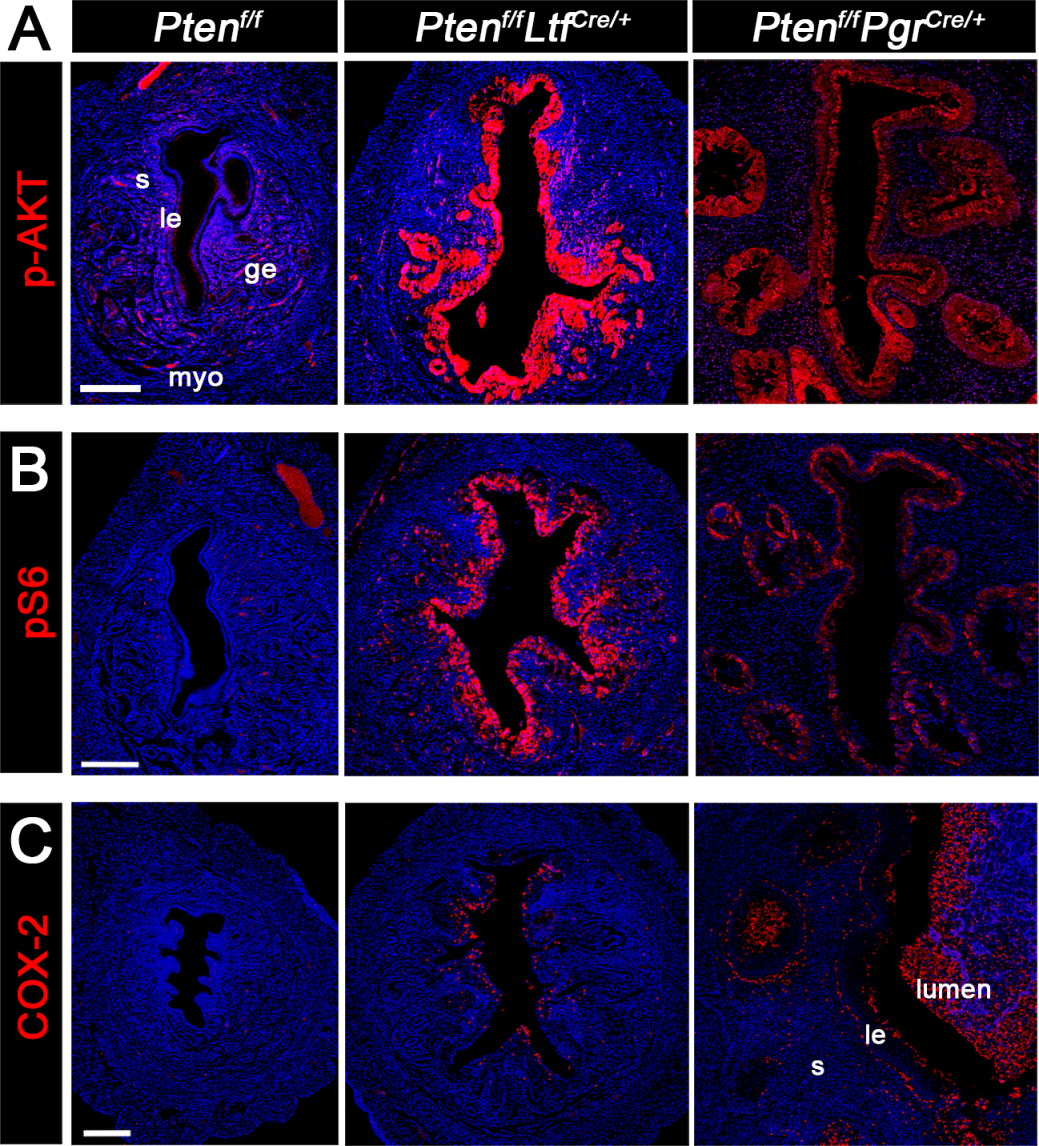
Differential expression of p-AKT, pS6, and COX-2 in the uteri from 3-month-old *Pten*^*f/f*^, *Pten*^*f/f*^*Ltf*^*Cre/+*^ and *Pten*^*f/f*^*Pgr*^*Cre/+*^ mice. A-C, Increased p-AKT, pS6 and COX-2 signals are observed in the epithelium of *Pten*^*f/f*^*Ltf*^*Cre/+*^ and *Pten*^*f/f*^*Pgr*^*Cre/+*^ uteri as compared to *Pten*^*f/f*^ uteri. In *Pten*^*f/f*^*Pgr*^*Cre/+*^ mice, COX-2 signals are also observed in debris inside the uterine lumen. Sections were counterstained with Hoechst (blue) to visualize the nuclei. Experiments were performed in three individual mice with the representative results presented. Scale bars, 200 μm. *le*, luminal epithelium; *ge*, glandular epithelium; *s*, stroma; *myo*, myometrium.

We compared the expression levels of p-AKT, pS6, and COX-2 in uteri of *Pten*^*f/f*^*Ltf*^*Cre/+*^ and *Pten*^*f/f*^*Pgr*^*Cre/+*^ females. Levels of p-AKT and pS6 are higher in *Pten*^*f/f*^*Ltf*^*Cre/+*^ uterine epithelia as compared to that in *Pten*^*f/f*^ uteri (Fig 2A and 2B). We have previously shown that COX-2 expression is associated with endometrial cancer progression, and inhibition of COX-2 slows down cancer development and progression [22]. Scattered signals of COX-2 are observed in *Pten*^*f/f*^*Ltf*^*Cre/+*^ epithelia, whereas COX-2 positive cells are widely distributed in *Pten*^*f/f*^*Pgr*^*Cre/+*^ epithelia and underneath stroma (Fig 2C). These results suggest that *Pten*^*f/f*^*Ltf*^*Cre/+*^ epithelial cells are less invasive as compared to *Pten*^*f/f*^*Pgr*^*Cre/+*^ epithelium. However, *Pten*^*f/f*^*Pgr*^*Cre/+*^ epithelial cells do not appear to undergo epithelial mesenchymal transition (EMT) as evident from staining of EMT markers E-cadherin (S6A Fig) and Desmin (S6B Fig) [23, 24].

### *Pten*^*f/f*^*Pgr*^*Cre/+*^ epithelia are stratified with progression of carcinoma

**Fig 3:**
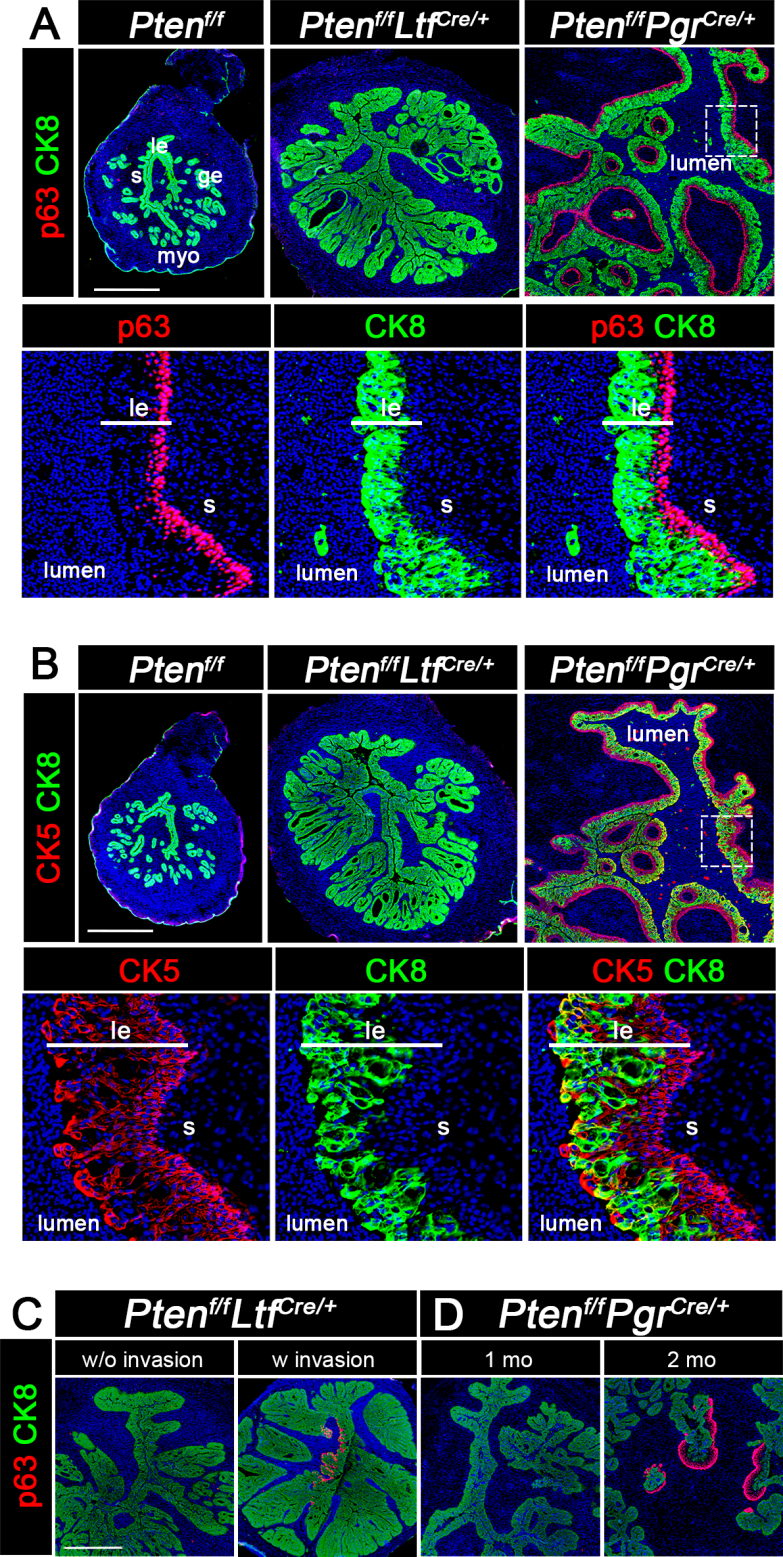
Expression of p63 and CK5 in *Pten*^*f/f*^*Pgr*^*Cre/+*^ uteri. A, Immunostaining of p63 and CK8 in uteri of 3-month-old *Pten*^*f/f*^, *Pten*^*f/f*^*Ltf*^*Cre/+*^ and *Pten*^*f/f*^*Pgr*^*Cre/+*^ mice. p63 is primarily detected in *Pten*^*f/f*^*Pgr*^*Cre/+*^ luminal epithelia. The framed area is shown at higher magnification in the lower panels. Noticeably, the expression of p63 and CK8 are mutually exclusive in the luminal epithelium. B, Immunostaining of CK5 and CK8 in uteri of 3-month-old *Pten*^*f/f*^, *Pten*^*f/f*^*Ltf*^*Cre/+*^ and *Pten*^*f/f*^*Pgr*^*Cre/+*^ mice. Expression pattern of CK5 and CK8 in *Pten*^*f/f*^*Pgr*^*Cre/+*^ luminal epithelium shows little overlap (yellow). C, Immunostaining of p63 in uteri of 4-month-old *Pten*^*f/f*^*Ltf*^*Cre/+*^ mice. p63 signals are observed in a limited number of luminal epithelial cells in *Pten*^*f/f*^*Ltf*^*Cre/+*^ mice with myometrium invasion. D, p63 expression becomes evident in *Pten*^*f/f*^*Pgr*^*Cre/+*^ uteri at 2 months of age. Nuclei are counterstained with Hoechst (blue). All experiments were performed in three mice. Scale bars, 200 μm. *le*, luminal epithelium; *ge*, glandular epithelium; *s*, stroma; *myo*, myometrium.

Previous studies showed that p63, a p53 homologue, is a marker of metaplastic differentiation, including basal/squamous differentiation and is found in stratified human tumors including EMC [25, 26]. Thus, we explored the expression of p63 in *Pten*^*f/f*^*Ltf*^*Cre/+*^ and *Pten*^*f/f*^*Pgr*^*Cre/+*^ uteri. As shown in Fig 3A, p63 is localized primarily in the basal layer of luminal epithelia of *Pten*^*f/f*^*Pgr*^*Cre/+*^ mice and surrounding glands at 3 months of age, while no p63 signal was observed in *Pten*^*f/f*^*Ltf*^*Cre/+*^ uteri at the same age. Notably, p63 positive cells express E-cadherin (S6C Fig), suggesting that these cells maintain epithelial characteristics. *Trp63* encodes multiple isoforms of p63, including full length TA isoforms with an acidic transactivation domain and ΔN isoforms lacking this domain [27]. Therefore, we used Western blotting analysis to assess the isoforms of p63 in uterine lysates of *Pten*^*f/f*^*PR*^*cre/+*^, *Pten*^*f/f*^*Ltf*^*cre/+*^ and respective littermate controls. The result shows that p63 in *Pten*^*f/f*^*PR*^*cre/+*^ epithelia is of TA isoform (S6D Fig).

Cytokeratin can also be used to distinguish simple or stratified epithelium [28]. CK8 is produced by simple epithelia [29], while CK5 is particularly expressed in the basal layer of stratified squamous epithelium. We found that the expression pattern of CK5 is similar to that of p63 (Fig 3B). Interestingly, co-staining of p63 or CK5 with CK8 identified that the expression pattern of p63/CK5 and CK8 are mutually exclusive in *Pten*^*f/f*^*Pgr*^*Cre/+*^ uteri (Fig 3A and 3B). These results suggest a potential relationship between p63 and EMC. Since two *Pten*^*f/f*^*Ltf*^*Cre/+*^ mice of 4 month old showed myometrial invasion, we examined the expression of p63 in these mice. Notably, p63 positive cells were observed in *Pten*^*f/f*^*Ltf*^*Cre/+*^ mice with myometrial invasion (Fig 3C). These results again suggest that expression of p63 correlates with EMC. To study the correlation between p63 and carcinoma progression, the expression of p63 in *Pten*^*f/f*^*Pgr*^*Cre/+*^ uteri was examined in uteri of 1 and 2-month old *Pten*^*f/f*^*Pgr*^*Cre/+*^ mice. *Pten*^*f/f*^*Pgr*^*Cre/+*^ uteri at 1 month of age are negative for p63 signal, but p63-positive cells appear underneath the CK8 positive luminal epithelium at 2 months of age (Fig 3D). These results provide evidence that stromal PTEN restrains epithelial stratification, and p63 serves as an indicator of EMC.

### Stromal PTEN prevents transformation of CAH to EMC by promoting apoptosis

**Fig 4:**
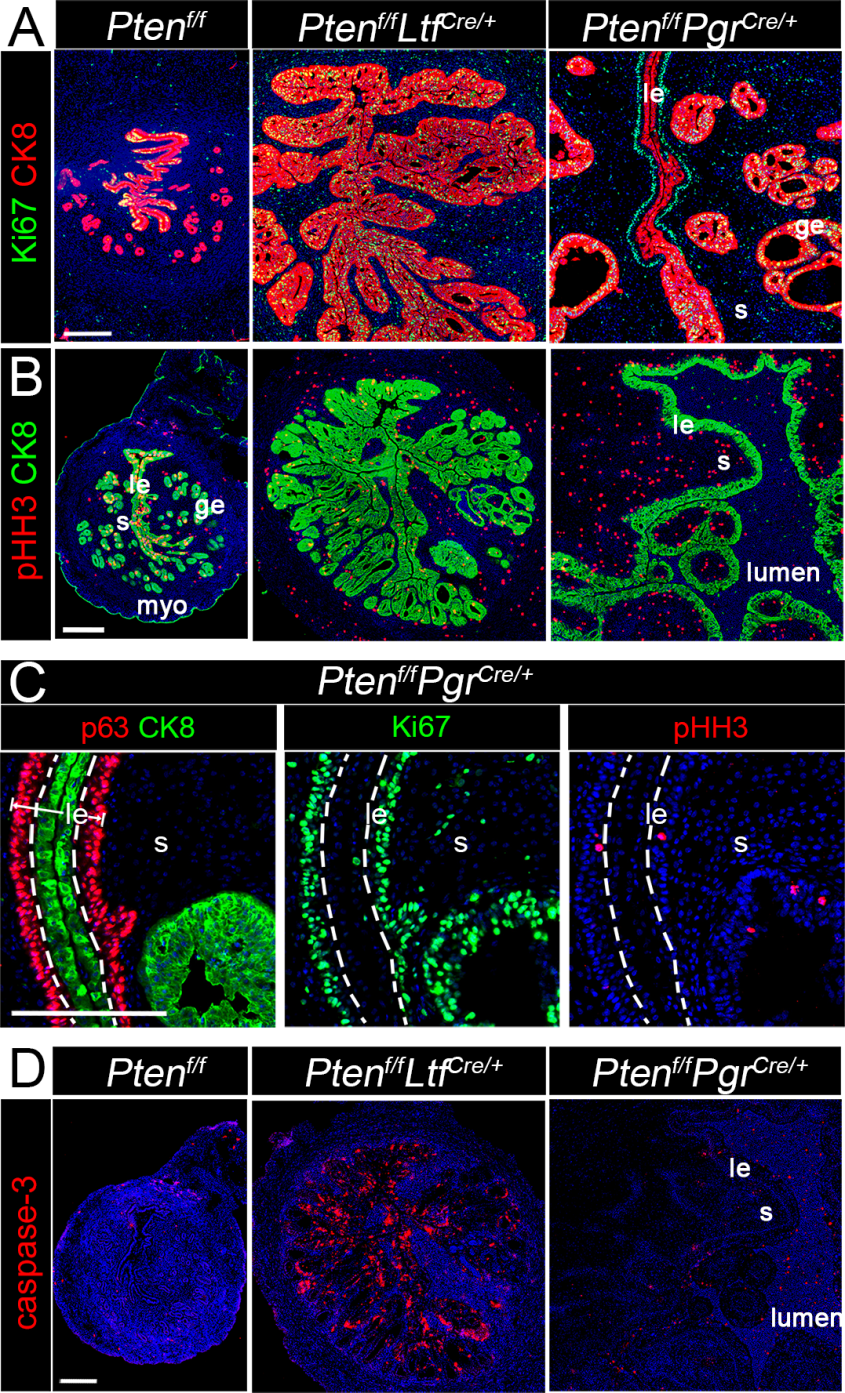
Proliferation and apoptosis in uteri of *Pten*^*f/f*^*Ltf*^*Cre/+*^ and *Pten*^*f/f*^*Pgr*^*Cre/+*^ mice at 3 months of age. A, Immunostaining of Ki67 and CK8. The expression of Ki67 show increased proliferation in *Pten*^*f/f*^*Ltf*^*Cre/+*^ and *Pten*^*f/f*^*Pgr*^*Cre/+*^ epithelium. Note that a line of Ki67^+^/CK8^-^ cells are under Ki67^-^/CK8^+^ epithelial cells. B, Expression of pHH3 in uteri of 3-month-old *Pten*^*f/f*^, *Pten*^*f/f*^*Ltf*^*Cre/+*^ and *Pten*^*f/f*^*Pgr*^*Cre/+*^ mice, respectively. C, Immunostaining of p63, CK8, Ki67 and pHH3 on consecutive serial sections. Almost all p63 positive cells are Ki67 positive in *Pten*^*f/f*^*Pgr*^*Cre/+*^ uteri. CK8 and p63 positive epithelial cells are separated by dotted lines. D, Immunostaining of Cleaved-caspase-3 in *Pten*^*f/f*^*Ltf*^*Cre/+*^ and *Pten*^*f/f*^*Pgr*^*Cre/+*^ uteri at 3 months of age. Nuclei are counterstained with Hoechst. Experiments were repeated in three mice, and representative images are presented. Scale bars, 200 μm. *le*, luminal epithelium; *ge*, glandular epithelium; *s*, stroma; *myo*, myometrium.

We explored the underlying mechanism preventing epithelial carcinoma by stromal PTEN. Since Ki67 positive cells are present at the leading edge of the tumor in *Pten*^*f/f*^*Pgr*^*Cre/+*^ uteri [9], we examined the distribution of Ki67-positive cells in *Pten*^*f/f*^*Ltf*^*Cre/+*^ uteri. As shown in Fig 4A, strong signals for Ki67 are present in both *Pten*^*f/f*^*Ltf*^*Cre/+*^ and *Pten*^*f/f*^*Pgr*^*Cre/+*^ uterine epithelium. Remarkably, Ki67 staining is more intense in the CK8-negative luminal epithelium. These results were corroborated by co-staining of CK8, p63, and Ki67 staining on the consecutive sections (Fig 4C). Ki67 signals are localized in p63-positive epithelia. The staining of phosphor-Histone H3 (pHH3) in *Pten*^*f/f*^*Pgr*^*Cre/+*^ uteri also showed similar expression pattern to that of Ki67 (Fig 4B and 4C). The results show that CK8 positive epithelial cells are proliferative in *Pten*^*f/f*^*Ltf*^*Cre/+*^ uteri, whereas epithelial cells in the p63-positive layer show cell proliferation in *Pten*^*f/f*^*Pgr*^*Cre/+*^ uteri. To better understand the turnover of epithelial cells in *Pten*^*f/f*^*Ltf*^*Cre/+*^ and *Pten*^*f/f*^*Pgr*^*Cre/+*^ uteri, we examined cell apoptosis by cleaved-Caspase 3 (caspase-3) immunostaining and observed increased cell population of caspase-3 positive cells in *Pten*^*f/f*^*Ltf*^*Cre/+*^ epithelia; the signal is limited in *Pten*^*f/f*^*Pgr*^*Cre/+*^ epithelia (Fig 4D). These results indicate that the PTEN-positive stroma in *Pten*^*f/f*^*Ltf*^*Cre/+*^ uteri restricts the epithelial hyperplasia by promoting apoptosis in hyperplastic epithelia, while *Pten* deletion in the stroma in *Pten*^*f/f*^*Pgr*^*Cre/+*^ uteri fails to prevent excessive proliferation and transform hyperplastic epithelial cells to EMC. Notably, *Pten*^*f/f*^*Amhr2*^*cre/+*^ uteri with *Pten* deletion in the stroma show apparently normal proliferation and apoptosis in epithelia (S7 Fig), suggesting stromal PTEN has limited impact on epithelial growth under normal physiological conditions.

Uterine cell proliferation and differentiation is regulated by ovarian hormones through ESR1 [30] and PR [31]. We examined the expression of these two nuclear receptors. The results show that the expression of ESR1 and PR is maintained in all major cell types in both *Pten*^*f/f*^*PR*^*cre/+*^ and *Pten*^*f/f*^*Ltf*^*cre/+*^ uteri (S8 Fig).

### Increased epithelial apoptosis is associated with elevated macrophage infiltration

**Fig 5:**
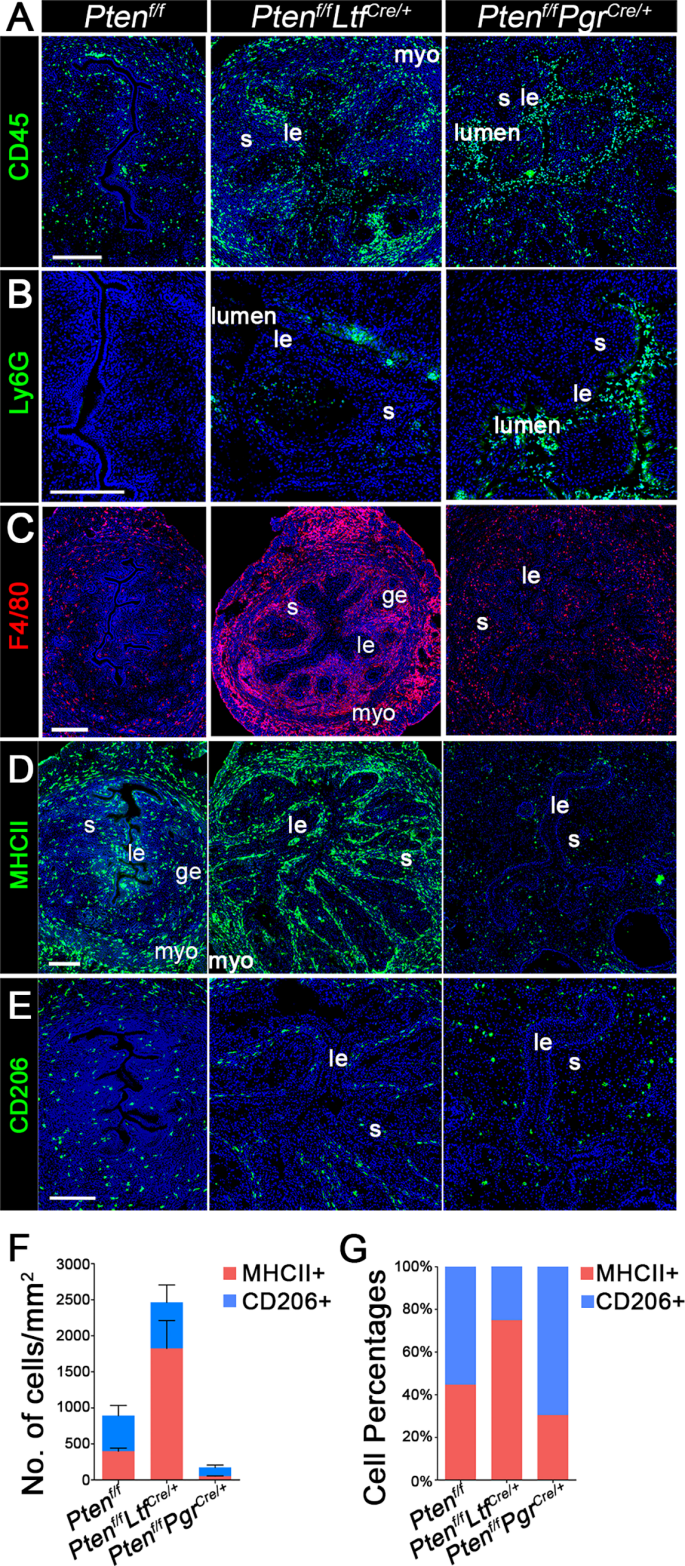
Increased epithelial apoptosis is associated with elevated macrophage infiltration in uteri of 3-month-old *Pten*^*f/f*^*Ltf*^*Cre/+*^ mice. A, Expression of CD45 shows distribution of all hematopoietic cells in the *Pten*^*f/f*^, *Pten*^*f/f*^*Ltf*^*Cre/+*^ and *Pten*^*f/f*^*Pgr*^*Cre/+*^ uteri. B, Cells positive for Ly6G, a marker of neutrophils, are increased in numbers in *Pten*^*f/f*^*Pgr*^*Cre/+*^ uteri as compared to those in *Pten*^*f/f*^ and *Pten*^*f/f*^*Ltf*^*Cre/+*^ uteri. C, More macrophages are observed in *Pten*^*f/f*^*Ltf*^*Cre/+*^ uteri as compared to those in *Pten*^*f/f*^*Pgr*^*Cre/+*^ uteri. D and E, Representative immunofluorescence images of MHCII, a marker for M1 macrophage, and CD206, a marker for M2 macrophage. Most macrophages in *Pten*^*f/f*^*Ltf*^*Cre/+*^ uteri are M1 macrophages. F, The numbers of MHCII and CD206 positive macrophages in stromal cells per square mm. G, the ratio of MHCII^+^/CD206^+^ cells is higher in *Pten*^*f/f*^*Ltf*^*Cre/+*^ uteri as compared to that in *Pten*^*f/f*^*Pgr*^*Cre/+*^ uteri. Experiments were performed in three mice, and representative images are presented. Scale bars, 200 μm. *le*, luminal epithelium; *ge*, glandular epithelium; *s*, stroma; *myo*, myometrium.

Given extensive apoptosis in *Pten*^*f/f*^*Ltf*^*Cre/+*^ uteri, we then asked if immune cells play a role in apoptotic cell clearance in *Pten*^*f/f*^*Ltf*^*Cre/+*^ uteri. First, we accessed the distribution of CD45-positive cells of hematopoietic origin. *Pten*^*f/f*^*Ltf*^*Cre/+*^ and *Pten*^*f/f*^*Pgr*^*Cre/+*^ uteri show increased population of immune cells in the uterus (Fig 5A); the weight of spleen, liver and thymus did not show many changes (S9 Fig). The uterine recruitment of immune cells suggests local inflammation. As previously reported that neutrophils are recruited in *Pten*^*f/f*^*Pgr*^*Cre/+*^ uteri [32], the population of Ly6G-positive cells, a marker of neutrophils, is much higher in *Pten*^*f/f*^*Pgr*^*Cre/+*^ uteri (Fig 5B). Interestingly, increased CD45-positive cells in *Pten*^*f/f*^*Ltf*^*Cre/+*^ are not neutrophils but macrophages, as shown by F4/80 staining (Fig 5C). The infiltration of different immune cells could be due to extensive apoptotic or metaplastic cells in *Pten*^*f/f*^*Ltf*^*Cre/+*^ and *Pten*^*f/f*^*Pgr*^*Cre/+*^ uteri respectively.

Two types of macrophages (M1 and M2) exhibit diverse phenotypes and functions. We examined the expression of MHCII and CD206, markers for M1 and M2 macrophages, respectively, to determine which subtypes contribute to increased macrophage population in *Pten*^*f/f*^*Ltf*^*Cre/+*^uteri. The results show that the number of MHCII-positive cells is much higher in *Pten*^*f/f*^*Ltf*^*Cre/+*^ uteri than that in *Pten*^*f/f*^*Pgr*^*Cre/+*^ (Fig 5D). However, no significant differences in CD206-positive M2 macrophages are observed (Fig 5E). The quantification of M1 versus M2 macrophages is shown in Fig 5F and 5G. Furthermore, F4/80-positive signals do not co-localize with Ki67 or caspase-3 signals (S10A and S10B Fig), suggesting that resident macrophages in the uterus do not proliferate but migrate from the circulation. These results suggest a potential role of macrophages in clearing apoptotic epithelial cells.

### TGFβ signaling in *Pten*^*f/f*^*Ltf*^*Cre/+*^ and *Pten*^*f/f*^*Pgr*^*Cre/+*^ uteri

**Fig 6:**
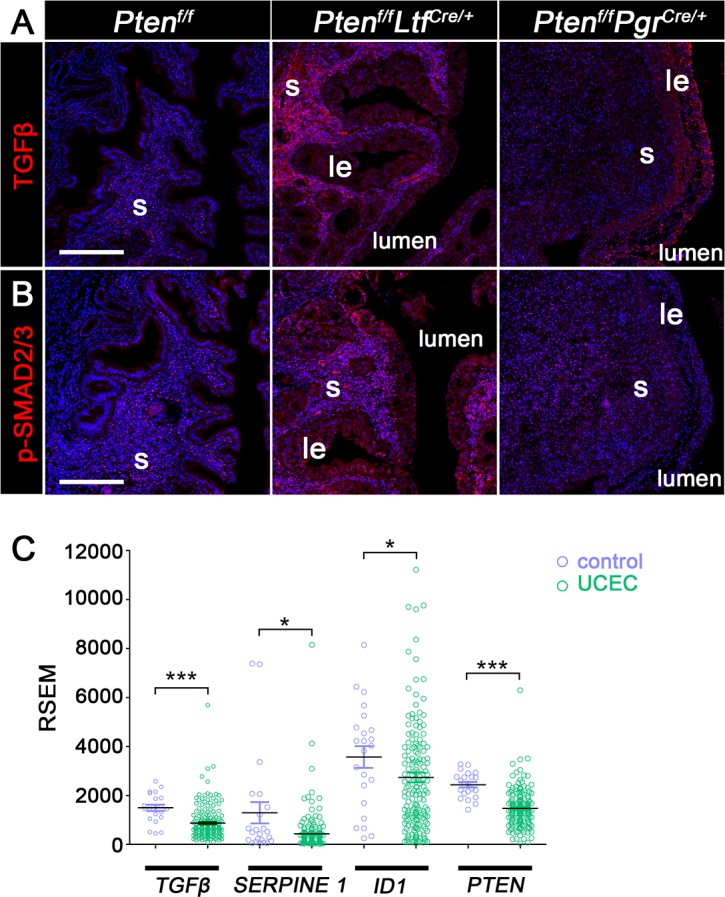
TGFβ signaling in the uteri of 3-month-old *Pten*^*f/f*^, *Pten*^*f/f*^*Ltf*^*Cre/+*^ and *Pten*^*f/f*^*Pgr*^*Cre/+*^ mice. A and B, Immunostaining of TGFβ and p-SMAD2/3 in *Pten*^*f/f*^*Ltf*^*Cre/+*^ and *Pten*^*f/f*^*Pgr*^*Cre*^/^+^ uteri. Nuclei are counterstained with Hoechst (blue). Experiments were performed in three mice, and representative images are presented. C. RNA levels of TGFβ and its target genes in patients with uterine corpus endometrial carcinoma (UCEC) from TCGA database. Scale bars, 200 **μ**m. le, luminal epithelium; ge, glandular epithelium; s, stroma; myo, myometrium. *, p<0.05; ***, p<0.01.

TGFβ signaling has been reported to have a dual function during the progression of carcinoma: cell-cycle arrest and apoptosis in the early-stage cancer and tumorigenesis at the late stage [33]. TGFβ signaling also inhibits epithelial stratification [17]. Using immunofluorescence, we observed distinct TGFβ signals in the stroma of *Pten*^*f/f*^*Ltf*^*Cre/+*^ uteri, whereas the signal is much lower in *Pten*^*f/f*^*Pgr*^*Cre/+*^ stroma, especially in the stroma surrounding the luminal epithelium (Fig 6A). The distribution of phosphorylated SMAD2/3 (p-SMAD2/3), a downstream effector, correlates with TGFβ signaling [34]. Consistent with TGFβ staining, the activation of p-SMAD2/3 is significantly lower in *Pten*^*f/f*^*Pgr*^*Cre/+*^ uteri (Fig 6B). These results suggest that stromal *Pten* potentially exerts its tumor suppressive role by upregulating TGFβ signaling.

To study if our findings have any relevance to human uterine corpus endometrial carcinoma (UCEC), we compared the RNA profile from patients with UCEC and controls that are available in RNA-seq dataset from The Cancer Genome Atlas (TCGA). Our analysis shows that the UCEC group has significantly lower levels of *PTEN* and *TGFβ* RNA, as well as *TGFβ*’s target genes, SERPINE1 and ID1, as compared to those in control tissues (Fig 6C). This is consistent with low TGFβ levels in *Pten*^*f/f*^*Pgr*^*Cre/+*^ uteri. (Fig 6C).

## Discussion

PTEN is considered as a tumor suppressor protein. *Pten* mutations are closely related to various types of tumorigenesis, especially type I EMC [35]. Our present and previous studies using cell specific deletion of *Pten* in the uterus provide evidence that absence of *Pten* in the epithelium, stroma and myometrium promptly produces EMC, while its deletion in the stroma and myometrium fail to generate EMC but transforms myometrial cells to adipocytes. Surprisingly, *Pten* deletion specifically in the epithelium primarily shows CAH.

The function of epithelial *Pten* has been studied using several approaches. Adenovirus was used to delete endometrial epithelial *Pten* by intraluminal injection [36], although a small percentage of endometrial stromal cells of adeno-*Cre* injected mice showed *Pten* deletion. Conditional *Pten* deletion using *Wnt7a-Cre* and *Ksp-Cre* in combination with *Pik3ca* mutation was also reported [37]. Combination of *Pten* deletion and Pik3ca mutation leads to carcinoma, while *Pik3ca* mutation alone showed no EMC or hyperplasia phenotype. Similar to the CAH phenotype in the uterus, *Pten* deletion leading to hyperplasia has been corroborated in several other different epithelial tissues besides the endometrial epithelium, such as urothelial cells, keratinocytes, prostatic epithelial cells, and lung epithelium [6]. Furthermore, the glandular epithelium specific *Pten* deletion also showed endometrial hyperplasia [38]. As *Pten* is deleted in both luminal and glandular epithelia in our *Ltf-iCre* model, definitive answers to distinguish the role of *Pten* in the luminal or glandular epithelium will require a luminal epithelium-specific deletion mouse model. It is also of interest to evaluate the uterine phenotype in stroma and glandular-deletion or stroma and luminal-deletion of *Pten* using a combination of *Cre* systems.

Our study with *Pten*^*f/f*^*Ltf*^*Cre/+*^ uteri suggests that stromal *Pten* restrains transition of hyperplasia to carcinoma. In contrast, the deficiency of this gene in three major uterine cell types (*Pten*^*f/f*^*Pgr*^*Cre/+*^) with rapid generation of EMC suggests that *Pten*-deleted stroma provides a more susceptible microenvironment for further deterioration of hyperplastic epithelium into EMC. In this regard, the role of endometrial stroma in EMC was reported using a stromal-specific Lkb1-deleted mouse model in which the loss of Lkb1 in the stroma was sufficient to initiate neoplasia [39]. A previous study also showed that EMC develops in uteri with epithelial modification in both *Pten* and *Pik3ca* [37]. In the mouse uterus, epithelial deletion of *Pten* alone is not sufficient to induce EMC. In human cancer specimens, PTEN is predominantly lost in the epithelium and maintained in the stroma [40]. EMC was also observed in a transplant model, in which a mixture of *Pten* deficient epithelial cells and WT stromal cells were transplanted under kidney capsule [40]. In spite of these findings, many questions still remain about the differences between human and mouse models of cancers.

The higher levels of pS6 signal in *Pten*^*f/f*^*Ltf*^*Cre/+*^ epithelia as compared with that in *Pten*^*f/f*^*Pgr*^*Cre/+*^ mice at 3 months of age suggest that activation of mTORC1 is closely associated with hyperplasia. Our previous study using mice with whole uterine deletion of Tsc1 also supports this conclusion [41]. Interestingly, mice with Tsc1 deletion in the stroma and myometrium also shows hyperplasia, suggesting the existence of unidentified paracrine signals from stromal influencing epithelial proliferation. Furthermore, our results with rapamycin (an inhibitor of mTORC1 signaling) suggest that inhibition of mTORC1 signaling could be an effective preventive strategy to combat endometrial hyperplasia and/or EMC. We have also shown that inhibition of upregulated COX-2 in the uterus of *Pten*^*f/f*^*Pgr*^*Cre/+*^ mice is reduced by a COX-2 inhibitor (Celecoxib) with attenuated EMC development [22]. In the present study, COX-2 is also induced in hyperplasic *Pten*^*f/f*^*Ltf*^*Cre/+*^ epithelia. By comparing the expression of COX-2 in *Pten*^*f/f*^*Ltf*^*Cre/+*^ and *Pten*^*f/f*^*Pgr*^*Cre/+*^ uteri, we found that the COX-2 level is much higher in *Pten*^*f/f*^*Pgr*^*Cre/+*^ uteri, suggesting hyperplasic cells are less invasive than cancerous cells.

The current study demonstrates that the expression of p63 is closely associated with EMC development. However, the role of p63 in uterine luminal epithelial stratification is still not clear. p63 plays multiple roles in development depending on different contexts [42]. p63 is required for establishing stratified epithelia perhaps by maintaining stem cell populations or triggering differentiation of simple epithelia into stratified epithelia [43, 44]. In humans, p63 is expressed in hyperplastic and metaplastic endometria [25]. It is possible that p63 suppresses epithelial metaplasia and prevents epithelia from further invading into the muscle layer, since the loss of p63 in tumor tissues is associated with more aggressive EMC [26]. p63-positive cells invade the area underneath p63-negative columnar cells and push them upward, which leads to the detachment of p63-negative cells [45]. However, we cannot rule out the possibility that p63 itself promotes EMC development.

TGFβ signaling appears to constrain hyperplastic *Pten*^*f/f*^*Ltf*^*Cre/+*^ epithelia from stratification toward tumorigenesis. TGFβ acts as a tumor suppressor in the epithelium [46] and restricts epithelial growth and early tumor development [47]. In mouse uteri, *TGFβr1* mRNA is detected mainly in the epithelia of *Pten*^*f/f*^*Pgr*^*Cre/+*^ uteri. SMAD2 is highly expressed in uterine epithelium at the proestrus phase. These results suggest a role for TGFβ in epithelial proliferation [48]. *Pten* and *TGFβr1* double knockout mice using *Pgr-Cre* driver show severe endometrial lesions with disrupted myometrial layers and pulmonary metastasis [49], suggesting a role for *TGFβr1* in cancer progression. Mice with uterine stromal *TGFβr1*-deletion using *Amhr2-Cre* show enhanced proliferation in both luminal and glandular epithelia [50], suggesting TGFβ signaling is involved in epithelial-stromal interactions. In this study, we observed PTEN levels are upregulated in the stroma of *Pten*^*f/f*^*Ltf*^*Cre/+*^ mice (Fig 1A) and is associated with heightened stromal TGFβ and pSMAD2/3 levels. In contrast, TGFβ levels are suppressed in *Pten*^*f/f*^*Pgr*^*Cre/+*^ stroma, indicating stromal TGFβ signaling may play a role in preventing epithelial tumorigenesis. Taken together, these data indicate that *Pten* expression in the stroma maintains stromal TGFβ expression, which perhaps limits epithelial growth.

TGFβ signaling plays a role in cell proliferation and apoptosis. In mouse uteri, reduced TGFβ signaling leads to loss of growth-inhibitory response, and constitutively activated TGFβr1 reduces glandular growth [51], suggesting an inhibitory role for TGFβ in epithelial proliferation. TGFβ signaling also plays a role in cell apoptosis. In polarized endometrial epithelial cells, TGFβ induces apoptosis via SMAD3 [52]. *Pten* knockdown blocks TGFβ-induced apoptosis and leads to increased cell proliferation. We observed intense cell apoptosis in *Pten*^*f/f*^*Ltf*^*Cre/+*^ mice compared to *Pten*^*f/f*^*Pgr*^*Cre/+*^ uteri, in which cell apoptosis is rarely seen. Interestingly, epithelial cell proliferation is not affected by changes in TGFβ signaling as evident by comparable numbers of Ki67 positive cells in *Pten*^*f/f*^*Ltf*^*Cre/+*^ and *Pten*^*f/f*^*Pgr*^*Cre/+*^. Given the role of TGFβ in apoptosis, the decreased levels of TGFβ and apoptosis in *Pten*^*f/f*^*Pgr*^*Cre/+*^ uteri suggest stromal PTEN-driven TGFβ prevents epithelial tumorigenesis by promoting epithelial cell apoptosis.

*Pten* deletion often leads to cancer *in situ* [53]. However, PTEN’s role in providing a microenvironment conducive to cancer progression is not clear. By comparing the phenotype of *Pten*^*f/f*^*Ltf*^*Cre/+*^ and *Pten*^*f/f*^*Pgr*^*Cre/+*^ mouse uteri, we show here that EMC development requires *Pten* deletion in both the stroma and epithelium. Our data also imply that stromal regulation of epithelial growth is mediated by TGFβ signaling. Our current study presents new insights into the role of *Pten* in the microenvironment for tumorigenesis.

## Materials and methods

### Mice

All mice were housed in the Cincinnati Children’s Hospital Medical Center Animal Care Facility in conformity with NIH and institutional guidelines. *Pten*^loxP/loxP^ mice (stock number 004597, 129S4/SvJae/BALB/cAnNTac) were obtained from the Jackson Laboratory (Sacramento, CA, USA). *Pten*^*f/f*^*Pgr*^*Cre/+*^ (129S4/SvJae/BALB/cAnNTac/C57BL/6) mice and *Ltf-iCre* mice (129/C57BL/6/albino B6) were generated as previously described [9, 13]. *Pten*^*f/f*^*Ltf*^*Cre/+*^ were generated by crossing *Pten*^loxP/loxP^ and *Ltf-iCre* mice. Littermate floxed mice were used as controls in all experiments. Uterine tissues from the diestrous stage were collected for experiments.

### Immunohistochemistry and immunofluorescence

For paraffin sections, tissues were fixed in Safefix (Thermo Fisher Scientific, Lafayette, CO, USA) and embedded in paraffin. After deparaffinization and hydration, sections (6 μm) were subjected to antigen retrieval by autoclaving in 0.01M sodium citrate solution (pH = 6) for 10 min. For frozen tissues, sections (12 μm) were fixed in 4% paraformaldehyde solution. Depending on the primary antibody (S1 Table), some sections were subjected to antigen retrieval by autoclaving in 0.01M sodium citrate solution (pH = 6) for 10 min. COX-2 and TGFβ antibodies were custom-made as previously described [54, 55]. For immunohistochemistry, Histostain-Plus kit (Invitrogen, Carlsbad, CA, USA) was used to visualize signals. Immunofluorescence was performed using secondary antibodies conjugated with Alexa 488 or Alexa 594 (Jackson ImmunoResearch, West Grove, PA, USA). Hematoxylin and Hoechst were used for counterstain in immunohistochemistry and immunofluorescence, respectively. For all images of pHH3 staining at lower magnification, the maximum filter of ImageJ was applied to the red staining channel for clear visibility.

### Quantification of macrophages

Numbers of M1 and M2 macrophages were calculated by counting MHCII and CD206 positive cells according to immunofluorescence staining. Sections from 3 different mice, and 4 fields per section have been evaluated.

### *In situ* hybridization

cRNA probes for *Pten* were generated by reverse RT-PCR followed by ^35^S-labeling using Sp6 or T7 RNA polymerases. Paraformaldehyde-fixed frozen sections (12 μm) were hybridized with ^35^S-labeled cRNA probes of *Pten* as previously described [9].

### Western blotting

Western blotting was performed as previously described [11]. Briefly, uterine protein samples from uteri at the diestrous stage were run on 10 or 12% SDS-PAGE gels depending on the molecular weights of proteins and transferred onto PVDF membranes. After blocking in 5% BSA for detection of phosphorylated protein, or in 10% non-fat milk for detection other proteins, membranes were blotted with antibodies to PTEN, p-AKT, AKT, pS6, S6, p63 and β-ACTIN. Signals were detected using ECL reagents (GE healthcare, Pittsburgh, PA, USA).

### TCGA analysis

RNAseq data were downloaded from TCGA data portal (https://tcga-data.nci.nih.gov/). RNAseq data from 176 UCEC cases and 23 controls were used for data analysis. Transcript-levels of genes were calculated using RNA-Seq by Expectation Maximization (RSEM) method.

### Statistical analysis

Data were analyzed by the Mann Whitney tests. P<0.05 was considered significant. Values are mean ± SEM.

## Supporting information

S1 Fig *Pten* is efficiently deleted in *Pten^f/f^Ltf^Cre/+^* epithelial cells. A, Genotyping of *Ltf-iCre*, *Pten* and Pten deletion (Δ5) in *Pten*^*f/f*^ and *Pten*^*f/f*^*Ltf*^*Cre/+*^ uteri. B, *In situ* hybridization of *Pten* in *Pten*^*f/f*^ and *Pten*^*f/f*^*Ltf*^*Cre/+*^ uteri. Experiments were performed in three individual mice with the representative results presented. Bar, 400 μm. *le*, luminal epithelium; *ge*, glandular epithelium; *s*, stroma; *myo*, myometrium.
(JPG)

S2 Fig Immunofluorescence of α-SMA and E-cad in uteri of *Pten^f/f^* and *Pten^f/f^Ltf^Cre/+^* mice at 6 and 12 months of age. Representative results from three individual mice are shown. Bar, 400 μm. le, luminal epithelium; ge, glandular epithelium; s, stroma; myo, myometrium.
(JPG)

S3 Fig Expression of CK8 and PTEN in uteri of *Pten^f/f^* and *Pten^f/f^Ltf^Cre/+^* mice at 4 months of age. A, Expression of CK8 in uteri of *Pten*^*f/f*^*Ltf*^*Cre/+*^ mice with myometrial invasion at 4 months of age. Images of *Pten*^*f/f*^ and *Pten*^*f/f*^*Ltf*^*Cre/+*^ uteri without myometrial invasion are presented in Fig 1B. B, Expression of PTEN in uteri of *Pten*^*f/f*^*Ltf*^*Cre/+*^ mice with or without myometrial invasion at 4 months of age. Arrowheads point to myometrial invasion in *Pten*^*f/f*^*Ltf*^*Cre/+*^ uteri. Experiments were performed in three mice. Representative result is shown. Bar, 400 **μm**. *le*, luminal epithelium; *ge*, glandular epithelium; *s*, stroma; *myo*, myometrium.
(JPG)

S4 Fig Expression of p-AKT, pS6, and COX-2 in uteri of *Pten^f/f^* and *Pten^f/f^Ltf^Cre/+^* mice at 2 and 3 months of age. The left panel represents uteri from 2-month-old mice, and the right panel shows uteri from 3-month-old mice. A-C, Immunohistochemistry of p-AKT, pS6 and COX-2 in uteri from 2- and 3-month-old *Pten*^*f/f*^*Ltf*^*Cre/+*^ and *Pten*^*f/f*^ mice. p-AKT and pS6 signals are detected in the epithelium of uteri from *Pten*^*f/f*^*Ltf*^*Cre/+*^ mice. COX-2 expression is increased in epithelial and surrounding stromal cells in *Pten*^*f/f*^*Ltf*^*Cre/+*^ mice. Sections are counterstained with hematoxylin. Experiments were repeated in three mice, and representative images are presented. Scale bar, 400 μm. *le*, luminal epithelium; *ge*, glandular epithelium; *s*, stroma; *myo*, myometrium.
(JPG)

S5 Fig Western blot analysis of PTEN, p-AKT, and pS6 using protein lysates from 2-month-old *Pten^f/f^* and *Pten^f/f^Ltf^Cre/+^* uteri. AKT, S6 and β-ACTIN serve as loading controls.
(JPG)

S6 Fig No EMT is observed in uteri of 3-month-old *Pten^f/f^*, *Pten^f/f^Ltf^Cre/+^* and *Pten^f/f^Pgr^Cre/+^* mice. A and B, Immunostaining of Desmin (a mesenchymal cell marker) and E-cad in uteri from *Pten*^*f/f*^, *Pten*^*f/f*^*Ltf*^*Cre/+*^ and *Pten*^*f/f*^*Pgr*^*Cre/+*^ mice at 3 months of age. Sections are counterstained with Hoechst. C, Immunofluorescence of p63 and epithelium marker E-cad in uteri of *Pten*^*f/f*^*Pgr*^*Cre/+*^ mice at 3 months of age. All p63 positive cells maintain E-cad expression. D, Western blotting of p63 in uteri from *Pten*^*f/f*^, *Pten*^*f/f*^*Ltf*^*Cre/+*^ and *Pten*^*f/f*^*Pgr*^*Cre/+*^ mice at 3 months of age. β-ACTIN serve as loading controls. Experiments were repeated in three mice, and a representative result is shown. Scale bar, 400 μm. *le*, luminal epithelium; *ge*, glandular epithelium; *s*, stroma; *myo*, myometrium.
(JPG)

S7 Fig Proliferation and apoptosis in uteri of *Pten^f/f^* and *Pten^f/f^Amhr2^Cre/+^* mice at 5 months of age. A and B, Immunostaining of Cleaved-caspase-3 and E-cad and Ki67 in uteri of 5-month-old *Pten*^*f/f*^ and *Pten*^*f/f*^*Amhr2*^*Cre/+*^ mice, respectively. Experiments were repeated in three mice with representative images presented. Scale bars, 400 μm. *le*, luminal epithelium; *ge*, glandular epithelium; *s*, stroma; *myo*, myometrium.
(JPG)

S8 Fig Expression of ESR1 and PR in uteri of 3-month-old *Pten^f/f^*, *Pten^f/f^Ltf^Cre/+^* and *Pten^f/f^Pgr^Cre/+^* mice. A, Immunostaining of ESR1 and E-cad. Nuclei are counterstained with Hoechst (blue). B, Immunofluorescence of PR and E-cad in uteri of 3-month-old *Pten*^*f/f*^, *Pten*^*f/f*^*Ltf*^*Cre/+*^ and *Pten*^*f/f*^*Pgr*^*Cre/+*^ mice. All experiments were performed in three mice. Scale bars, 400 μm. *le*, luminal epithelium; *ge*, glandular epithelium; *s*, stroma; *myo*, myometrium.
(JPG)

S9 Fig Wet weights of spleen, liver and thymus show no changes between *Pten^f/f^* and *Pten^f/f^Ltf^Cre/+^* mice. (JPG)

S10 Fig Immunostaining of F4/80 with Ki67 or Cleaved-caspase-3 in uteri of 3-month-old *Pten^f/f^Ltf^Cre/+^* mice. A, Immunofluorescence of F4/80 and Ki67 shows no colocalization. B, Immunofluorescence of Cleaved-caspase-3 and F4/80. Scale bars, 200 μm. *le*, luminal epithelium; *s*, stroma; *myo*, myometrium.
(JPG)

S1 Table List of antibodies used for immunohistochemistry, immunofluorescence and western blotting. (XLSX)
