# Supplementary figures and images for: The uterine epithelial loss of Pten is inefficient to induce endometrial cancer with intact stromal Pten
Source: PLoS Genet. 2018 Aug 24;14(8):e1007630. doi: 10.1371/journal.pgen.1007630 (PMC6126871; doi:10.1371/journal.pgen.1007630)

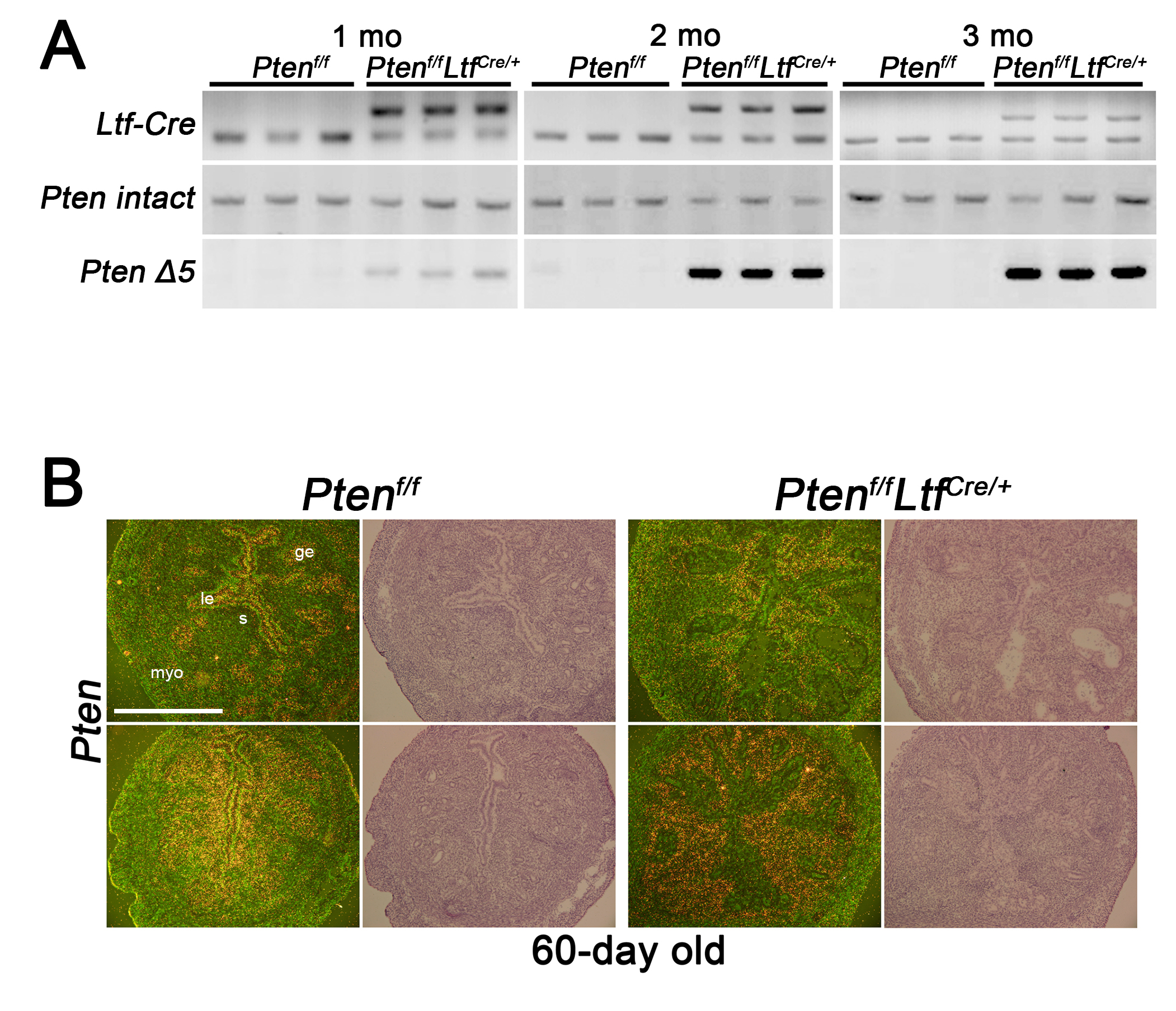

Supplement: S1 Fig — A, Genotyping of Ltf-iCre, Pten and Pten deletion (Δ5) in Ptenf/f and Ptenf/fLtfCre/+ uteri. B, In situ hybridization of Pten in Ptenf/f and Ptenf/fLtfCre/+ uteri. Experiments were performed in three individual mice with the representative results presented. Bar, 400 μm. le, luminal epithelium; ge, glandular epithelium; s, stroma; myo, myometrium. (JPG) [file pgen.1007630.s001.jpg]

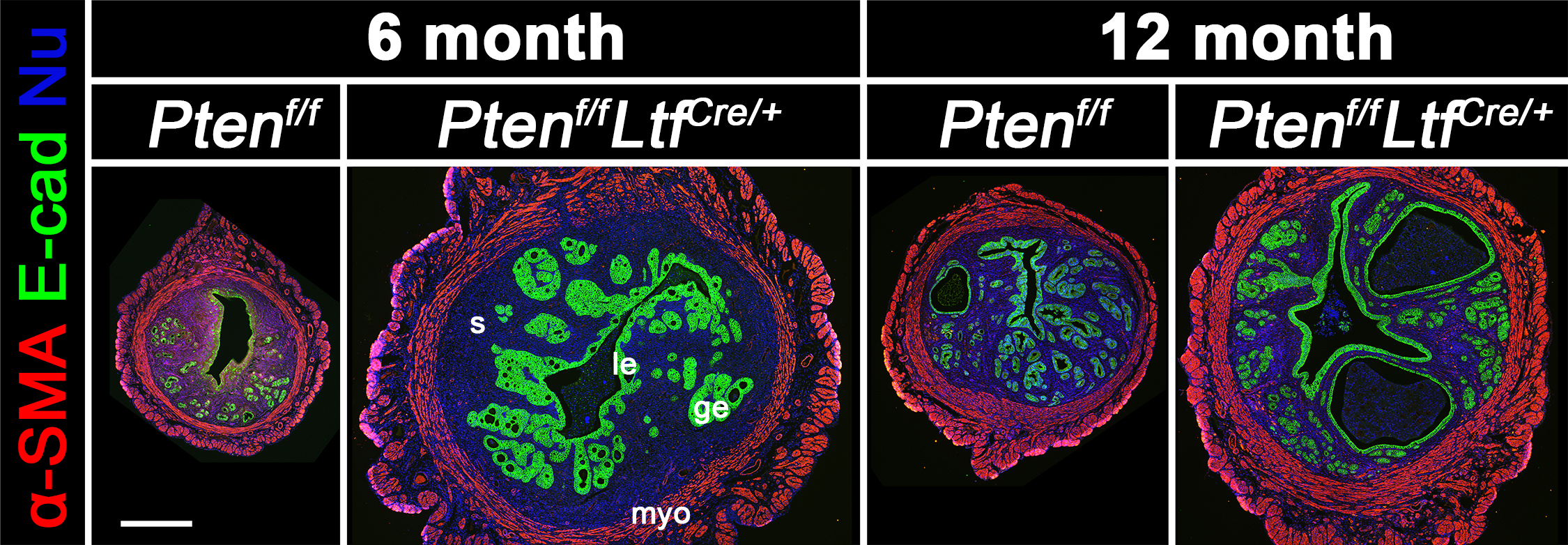

Supplement: S2 Fig — Representative results from three individual mice are shown. Bar, 400 μm. le, luminal epithelium; ge, glandular epithelium; s, stroma; myo, myometrium. (JPG) [file pgen.1007630.s002.jpg]

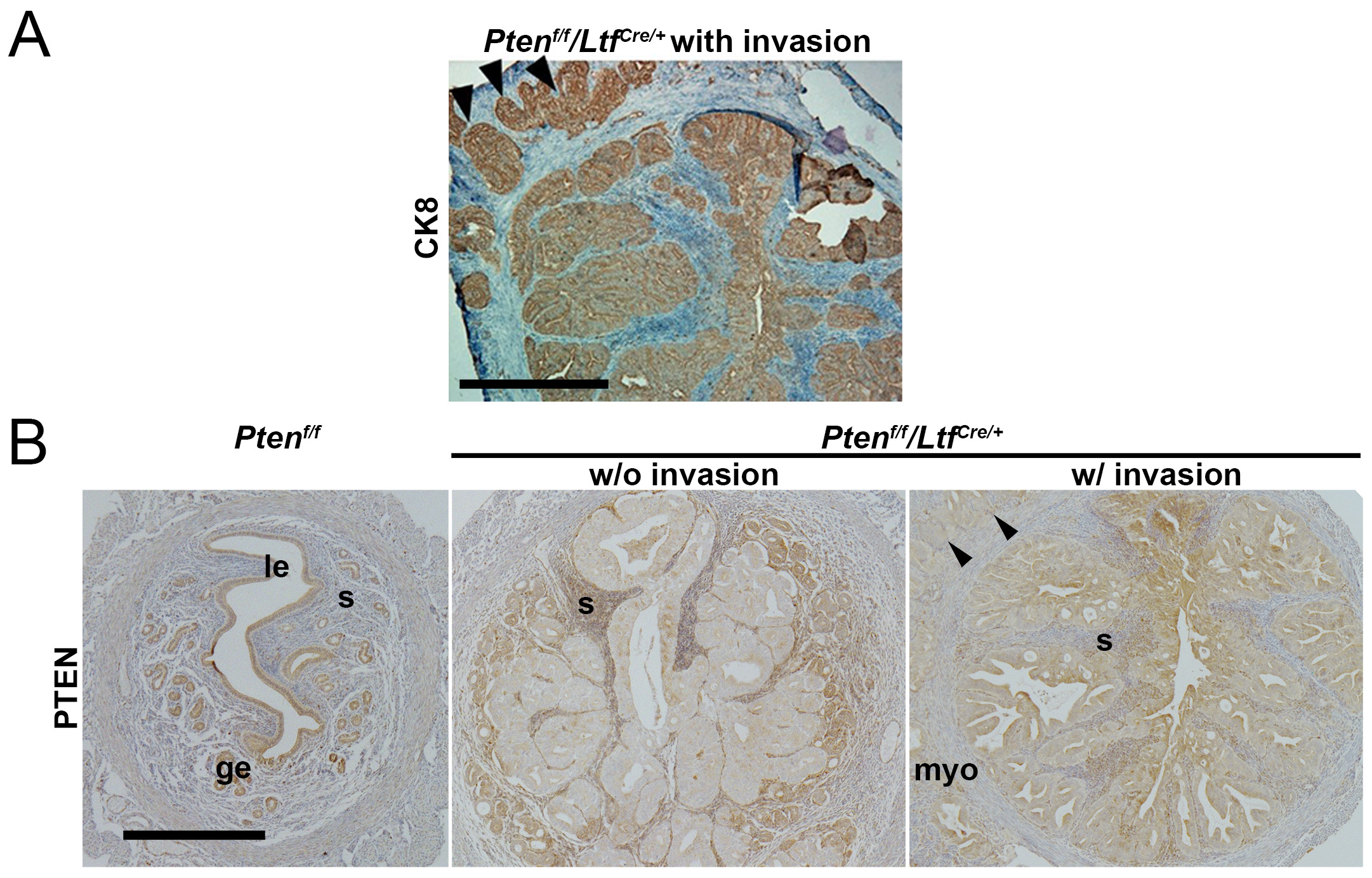

Supplement: S3 Fig — A, Expression of CK8 in uteri of Ptenf/fLtfCre/+ mice with myometrial invasion at 4 months of age. Images of Ptenf/f and Ptenf/fLtfCre/+ uteri without myometrial invasion are presented in Fig 1B. B, Expression of PTEN in uteri of Ptenf/fLtfCre/+ mice with or without myometrial invasion at 4 months of age. Arrowheads point to myometrial invasion in Ptenf/fLtfCre/+ uteri. Experiments were performed in three mice. Representative result is shown. Bar, 400 μm. le, luminal epithelium; ge, glandular epithelium; s, stroma; myo, myometrium. (JPG) [file pgen.1007630.s003.jpg]

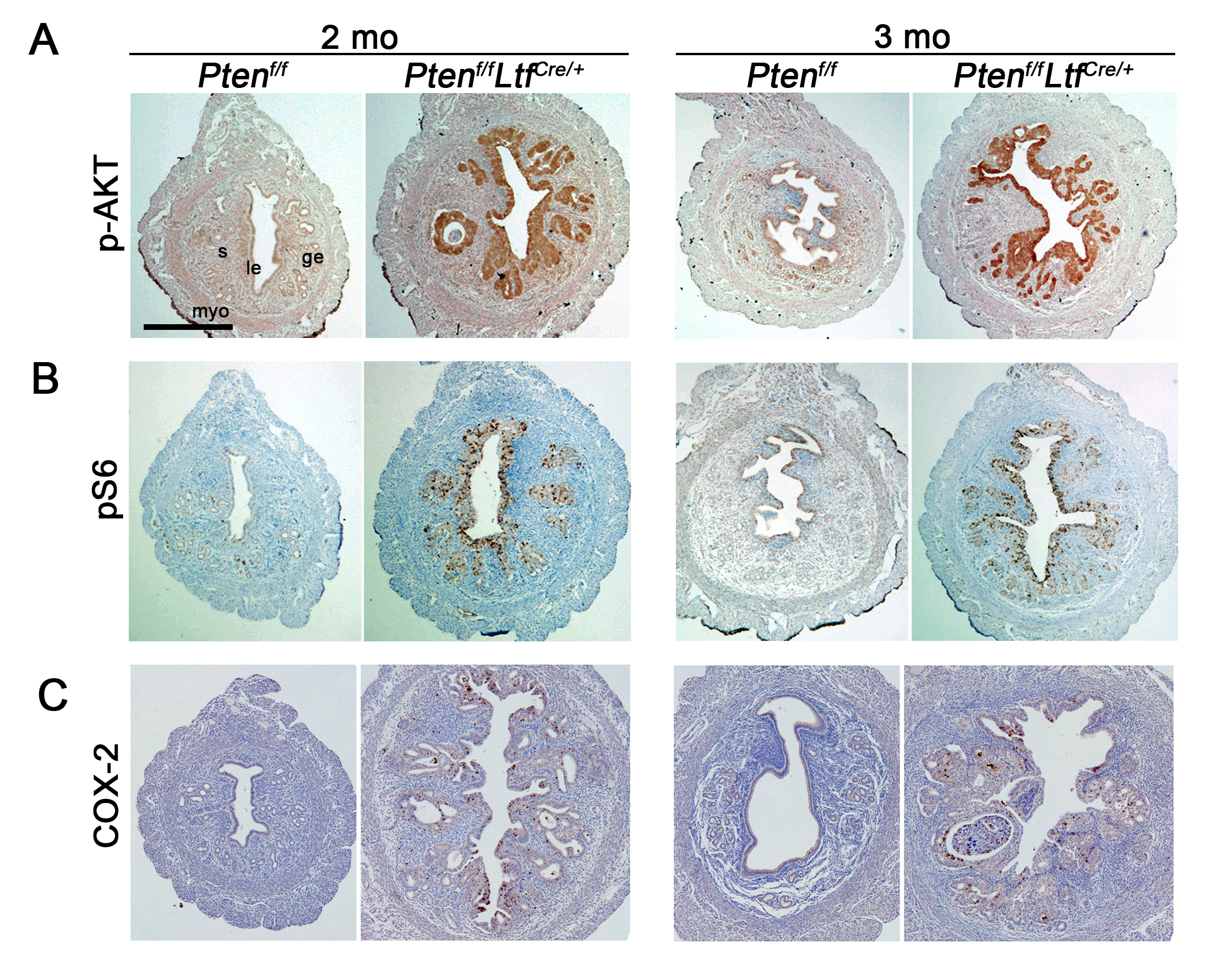

Supplement: S4 Fig — The left panel represents uteri from 2-month-old mice, and the right panel shows uteri from 3-month-old mice. A-C, Immunohistochemistry of p-AKT, pS6 and COX-2 in uteri from 2- and 3-month-old Ptenf/fLtfCre/+ and Ptenf/f mice. p-AKT and pS6 signals are detected in the epithelium of uteri from Ptenf/fLtfCre/+ mice. COX-2 expression is increased in epithelial and surrounding stromal cells in Ptenf/fLtfCre/+ mice. Sections are counterstained with hematoxylin. Experiments were repeated in three mice, and representative images are presented. Scale bar, 400 μm. le, luminal epithelium; ge, glandular epithelium; s, stroma; myo, myometrium. (JPG) [file pgen.1007630.s004.jpg]

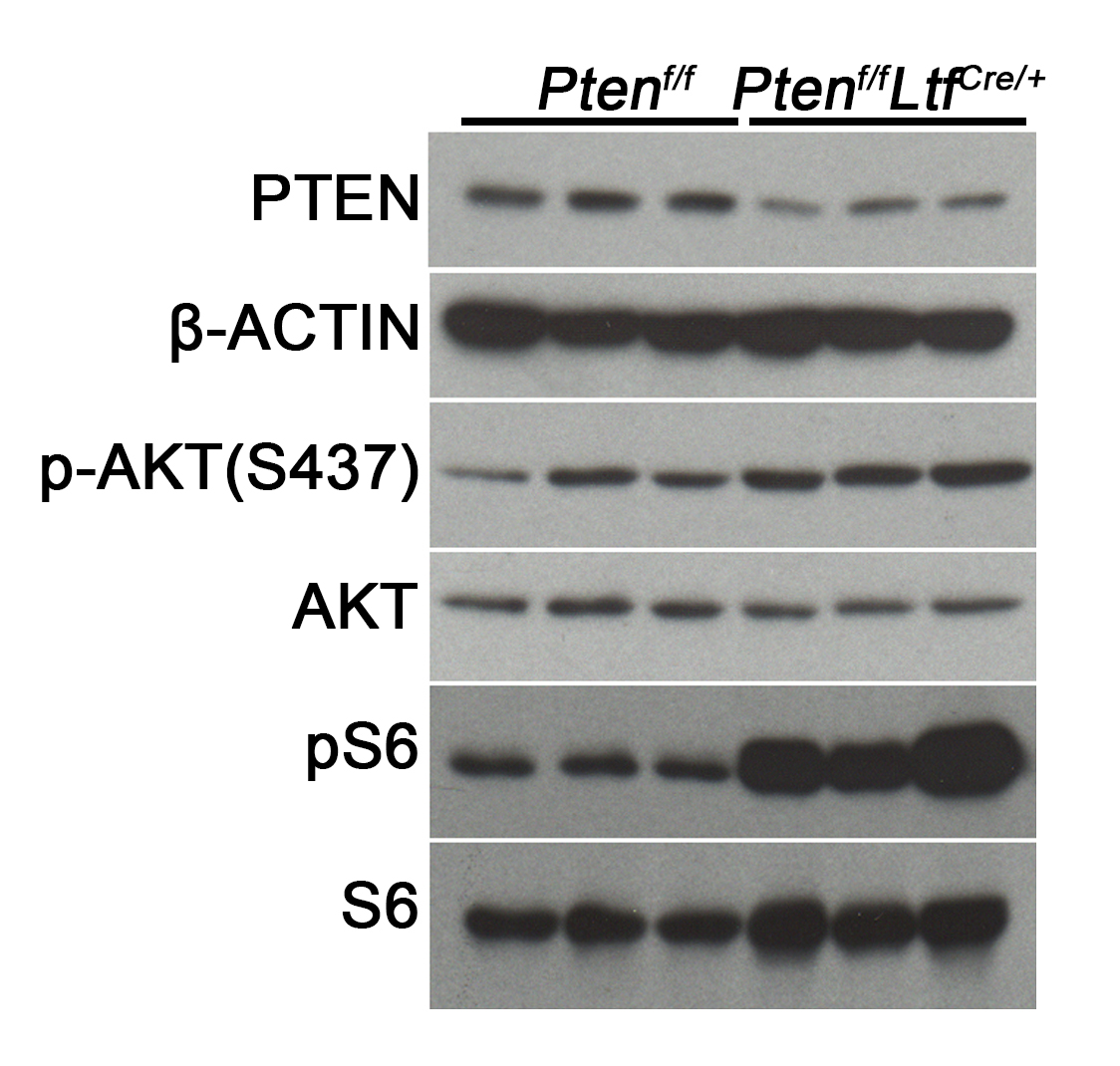

Supplement: S5 Fig — AKT, S6 and β-ACTIN serve as loading controls. (JPG) [file pgen.1007630.s005.jpg]

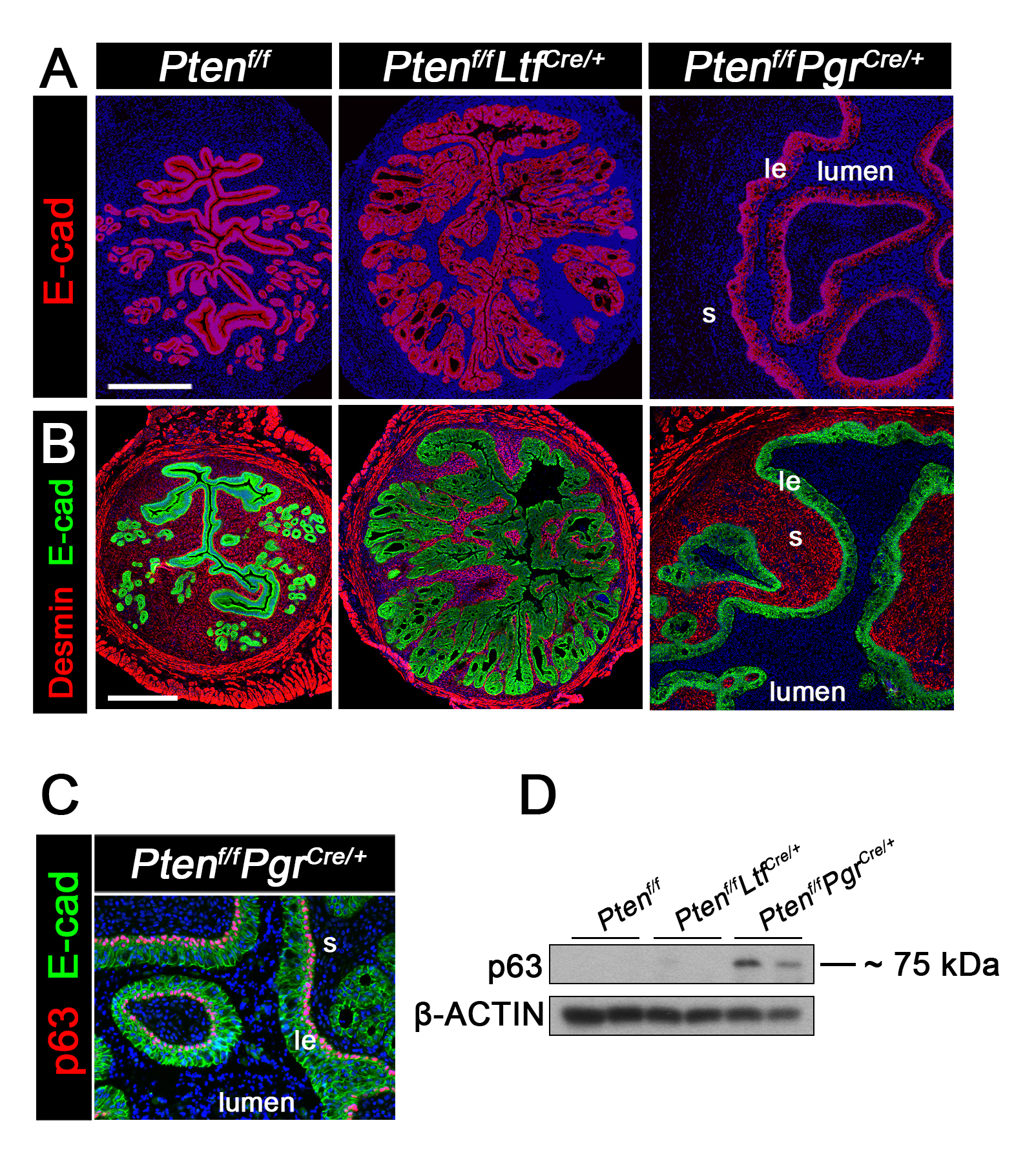

Supplement: S6 Fig — A and B, Immunostaining of Desmin (a mesenchymal cell marker) and E-cad in uteri from Ptenf/f, Ptenf/fLtfCre/+ and Ptenf/fPgrCre/+ mice at 3 months of age. Sections are counterstained with Hoechst. C, Immunofluorescence of p63 and epithelium marker E-cad in uteri of Ptenf/fPgrCre/+ mice at 3 months of age. All p63 positive cells maintain E-cad expression. D, Western blotting of p63 in uteri from Ptenf/f, Ptenf/fLtfCre/+ and Ptenf/fPgrCre/+ mice at 3 months of age. β-ACTIN serve as loading controls. Experiments were repeated in three mice, and a representative result is shown. Scale bar, 400 μm. le, luminal epithelium; ge, glandular epithelium; s, stroma; myo, myometrium. (JPG) [file pgen.1007630.s006.jpg]

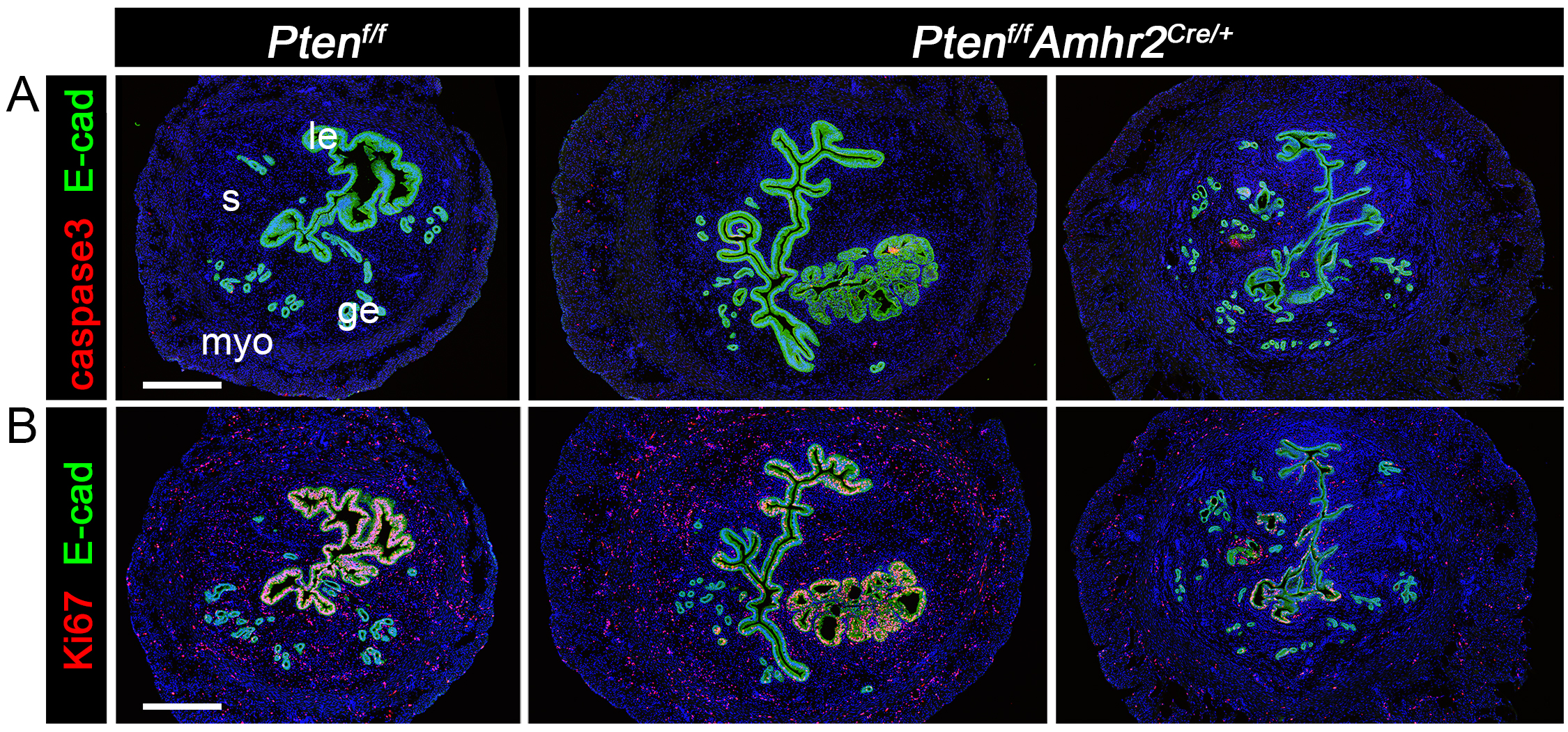

Supplement: S7 Fig — A and B, Immunostaining of Cleaved-caspase-3 and E-cad and Ki67 in uteri of 5-month-old Ptenf/f and Ptenf/fAmhr2Cre/+ mice, respectively. Experiments were repeated in three mice with representative images presented. Scale bars, 400 μm. le, luminal epithelium; ge, glandular epithelium; s, stroma; myo, myometrium. (JPG) [file pgen.1007630.s007.jpg]

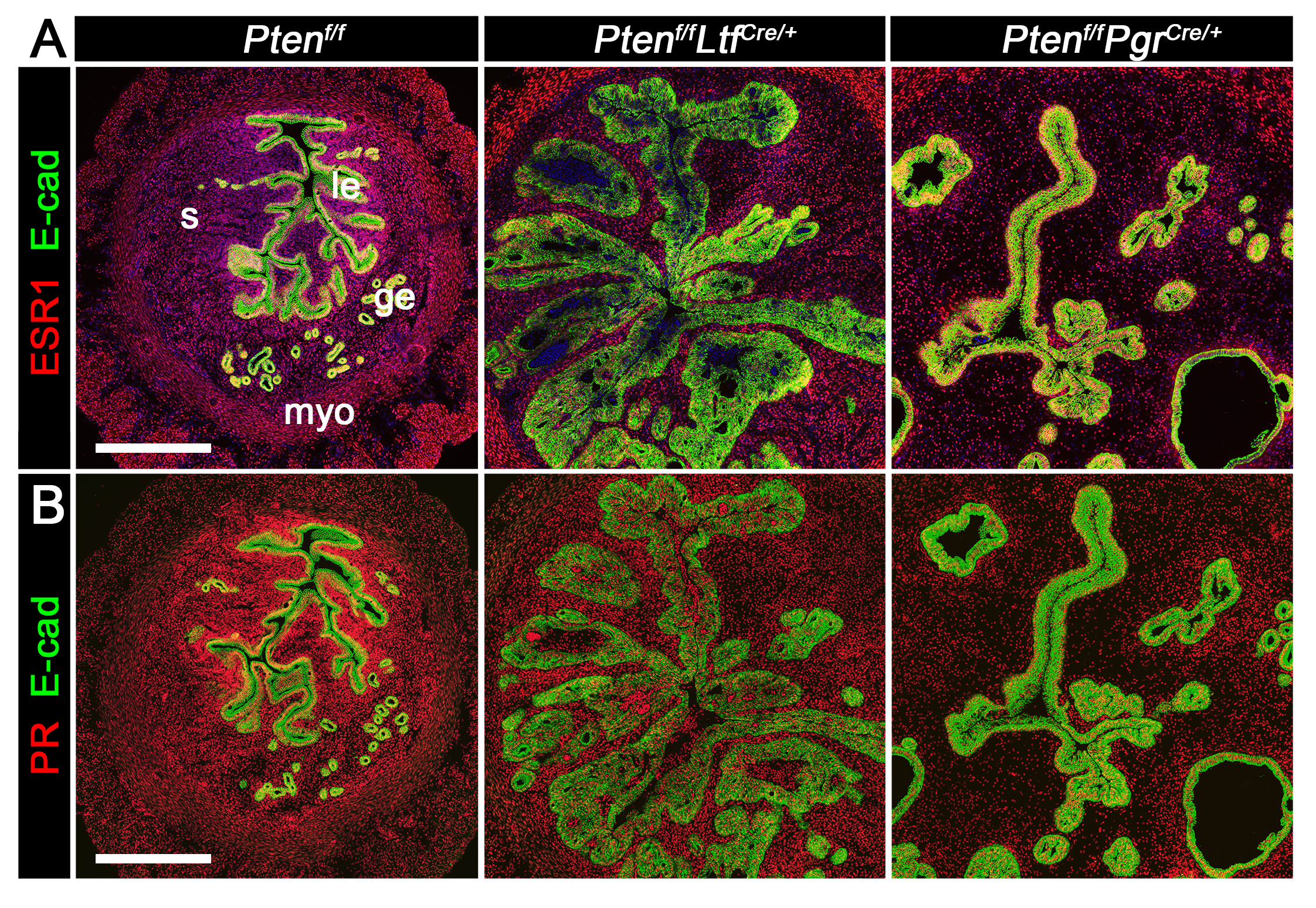

Supplement: S8 Fig — A, Immunostaining of ESR1 and E-cad. Nuclei are counterstained with Hoechst (blue). B, Immunofluorescence of PR and E-cad in uteri of 3-month-old Ptenf/f, Ptenf/fLtfCre/+ and Ptenf/fPgrCre/+ mice. All experiments were performed in three mice. Scale bars, 400 μm. le, luminal epithelium; ge, glandular epithelium; s, stroma; myo, myometrium. (JPG) [file pgen.1007630.s008.jpg]

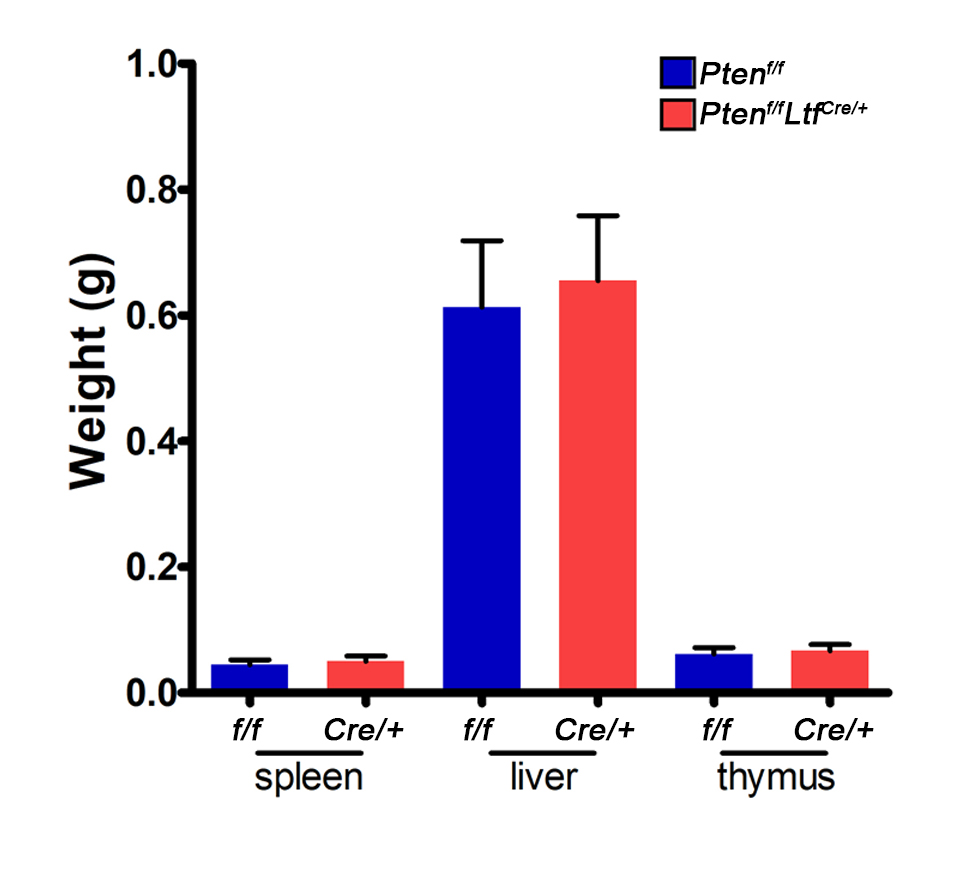

Supplement: S9 Fig — (JPG) [file pgen.1007630.s009.jpg]

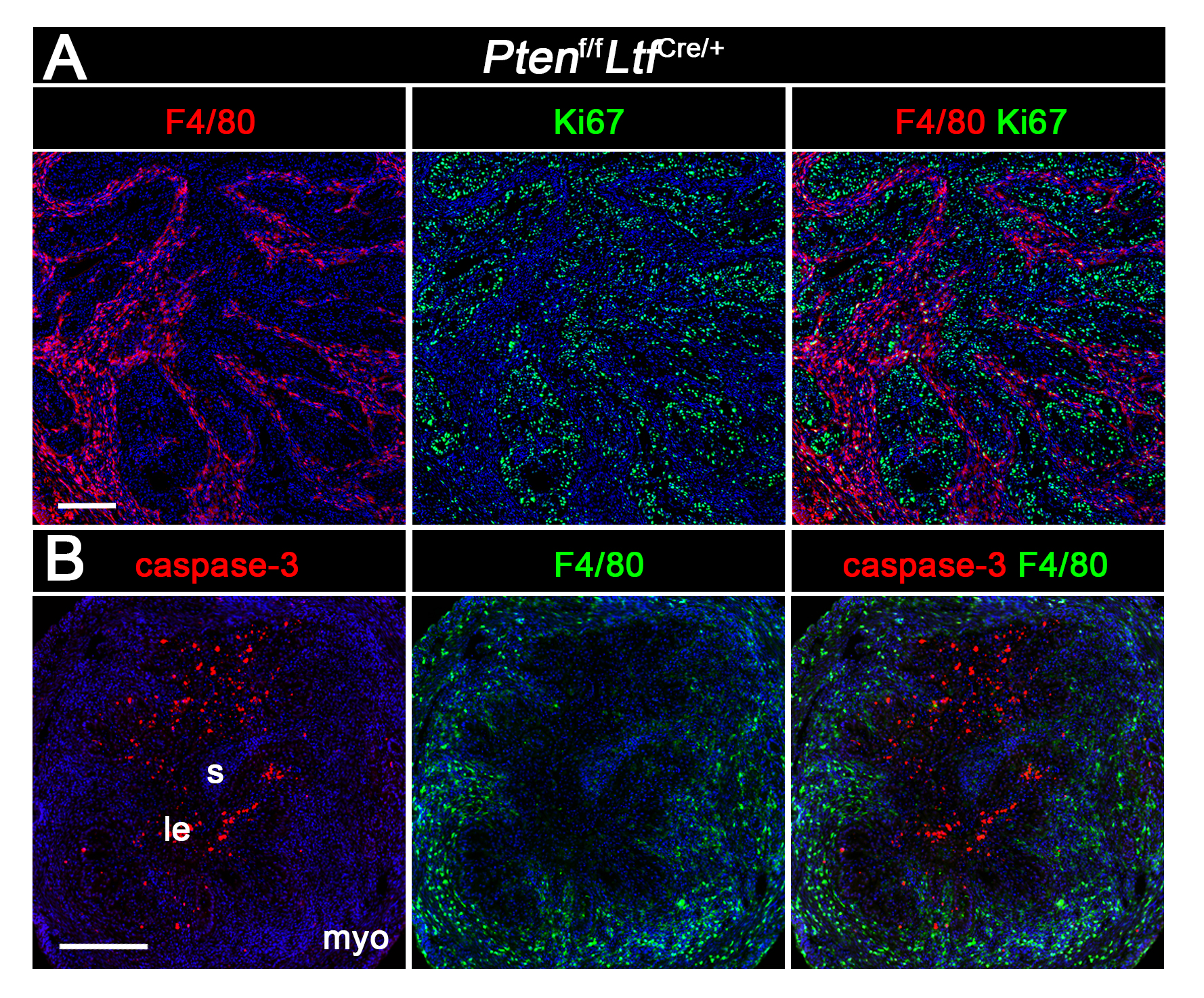

Supplement: S10 Fig — A, Immunofluorescence of F4/80 and Ki67 shows no colocalization. B, Immunofluorescence of Cleaved-caspase-3 and F4/80. Scale bars, 200 μm. le, luminal epithelium; s, stroma; myo, myometrium. (JPG) [file pgen.1007630.s010.jpg]
